# Spike 1 protein of SARS-CoV-2 induces endothelial inflammation and vascular dysfunction through interferon ISG15-dependent mechanisms

**DOI:** 10.1093/cvr/cvag111

**Published:** 2026-05-18

**Authors:** Francisco J Rios, Augusto C Montezano, Livia L Camargo, Rheure A Lopes, Ana B García-Redondo, Elihu Aranday-Cortes, Ana M Briones, John McLauchlan, Rhian M Touyz

**Affiliations:** Department of Medicine, Research Institute of the McGill University Health Centre, 1001, boul Décarie, ES1.5066.6, Montreal, Québec H4A 3J1, Canada; School of Medicine, Pharmacy and Biomedical Sciences, University of Portsmouth, St Michael’s building, White Swan Rd, Portsmouth PO1 2DT, UK; Department of Medicine, Research Institute of the McGill University Health Centre, 1001, boul Décarie, ES1.5066.6, Montreal, Québec H4A 3J1, Canada; Department of Medicine, Research Institute of the McGill University Health Centre, 1001, boul Décarie, ES1.5066.6, Montreal, Québec H4A 3J1, Canada; Institute of Cardiovascular and Medical Sciences, British Heart Foundation (BHF), Glasgow Cardiovascular Research Centre, University of Glasgow, Glasgow, UK; Departmento de Fisiología, Facultad de Medicina, Universidad Autónoma de Madrid, Madrid, Spain; Department of Physiology, Universidad Autónoma de Madrid, Instituto Investigación Hospital Universitario La Paz (IdiPaz), Madrid, Spain; Centro de Investigación Biomédica en Red de Enfermedades Cardiovasculares, Madrid, Spain; Medical Research Council University of Glasgow Centre for Virus Research, Glasgow, UK; Centro de Investigación Biomédica en Red de Enfermedades Cardiovasculares, Madrid, Spain; Departamento de Farmacología, Universidad Autónoma de Madrid, Instituto de Investigación Hospital La Paz, Madrid, Spain; Medical Research Council University of Glasgow Centre for Virus Research, Glasgow, UK; Department of Medicine, Research Institute of the McGill University Health Centre, 1001, boul Décarie, ES1.5066.6, Montreal, Québec H4A 3J1, Canada; Departments of Medicine and Department of Family Medicine, McGill University, 1001, boul Décarie, ES1.5066.6, Montréal, Québec H4A 3J1, Canada

**Keywords:** IFNα, IFNλ, Endothelial cells, COVID-19, SARS-CoV-2, Vascular dysfunction

## Abstract

**Aims:**

Interferon (IFN) alpha (IFNα) and lambda3 (IFNλ3) constitute first-line responses of immunity against severe acute respiratory syndrome coronavirus 2 (SARS-CoV-2) infection by increasing interferon-stimulated genes (ISGs). Prolonged IFN production may exacerbate inflammation, contributing to endotheliitis and vascular dysfunction in coronavirus disease 2019 (COVID-19). We investigated whether spike protein S1 (SP1) of SARS-CoV-2 via IFN influences inflammation in human vascular and lymphatic endothelial cells (ECs) and whether these processes contribute to vascular dysfunction in the context of hypertension. We focused on ISG15, a crucial immune protein that is also implicated in hypertension-associated vascular injury.

**Methods and results:**

Exposure of microvascular ECs to SP1 of SARS-CoV-2-induced expression of ISGs: ISG15, MX1, and IFIT1. These effects were potentiated by IFNs and reduced by ADAM17 and STAT1 inhibition and genetic inhibition of IFNα and beta receptor subunit 1 (IFNAR1). In microvascular ECs IFNλ3 and IFNα increased expression of ISGs, TMPRSS2, ADAM17, production of pro-inflammatory mediators (tumor necrosis factor [TNF]α, interleukin [IL]-6, plasminogen activator inhibitor [PAI]-1) and reduced phosphorylation of eNOS (Ser1177). In pulmonary, lymphatic, and aortic ECs, IFNα, but not IFNλ3, increased expression of ISGs and IL-6. To explore the relevance in intact vessels, effects of IFNs were studied in isolated micro-vessels from wildtype (WT), hypertensive and ISG15^−/−^ mice. IFNα, IFNλ3, and SP1 reduced endothelium-dependent relaxation in WT vessels, whereas IFNα increased contraction in vessels from hypertensive mice. Vascular dysfunction induced by IFNα, IFNλ3 or spike protein was abrogated in vessels from ISG15−/− mice.

**Conclusion:**

SP1 and IFNs synergically increase EC expression of ISGs through ADAM17. IFNλ3 and IFNα promote endothelial inflammation and vascular dysfunction through ISG15. These processes may play a role in the endotheliopathy and vascular damage associated with SP1 and might contribute to cardiovascular sequelae, including hypertension, of SARS-CoV-2 infection.

## Graphical Abstract

**Graphical Abstract:**
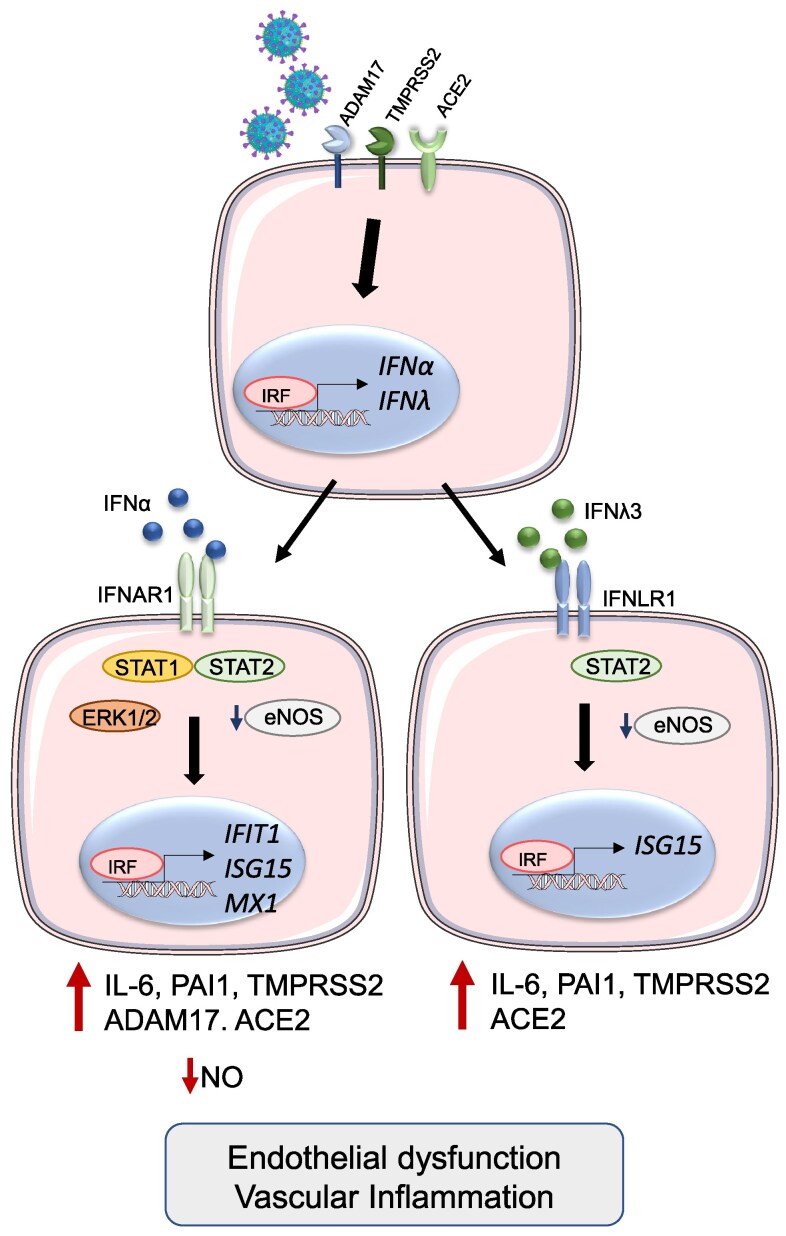
**Schematic demonstrating the link between spike protein S1 (SP1), IFNs, and endothelial cells.** SP1 interacts with endothelial cells and induces expression of Interferon-Stimulated Genes (ISGs) [ISG15, MX1, IFIT1], IFNλ3 and IFNα. IFNλ3 and IFNα, produced in viral-infected cell, are associated with reduced eNOS activation, endothelial cell inflammation, and endothelial dysfunction. Activation of IFNλ3/IFNAR1 and IFNα/IFNLR1, differentially activates intracellular signalling pathways, as demonstrated in the figure. Effects of SP1, IFNλ3 and IFNα are mediated via ISG15-dependent mechanisms.

**Time of primary review: 84 days**

## 1. Introduction

Interferon-alpha (IFNα) and IFN-lambda (IFNλ) are produced by different cell populations and constitute the first major line of innate immunity defence against virus infection and are produced by severe acute respiratory syndrome coronavirus 2 (SARS-CoV-2)-infected cells.^1^ Although they have affinity for different receptors, IFNα and IFNλ exhibit similarities in intracellular signalling by activating signal transducer and activator of transcription-1 (STAT1), STAT2 and IFN regulatory factor 9 (IRF9), leading to increased expression of a cascade of interferon-stimulated genes (ISGs), including ISG15, required for the inflammatory response and anti-viral activity.^1^ IFNα interacts with IFN alpha and beta receptor subunit 1/2 (IFNAR1)/IFNAR2, which is ubiquitously expressed and induces inflammation by promoting cytokine and chemokine production and recruitment of inflammatory cells to tissues. In contrast, IFNλ3 exerts its effects through a dimeric (IFN Lambda Receptor 1/Interleukin-10 Receptor Subunit Beta [IFNLR1/IL10RB] complex, highly expressed in epithelial and immune cells.^1,2^ Besides the important antiviral effects of IFN, the therapeutic use of IFN-based therapy in coronavirus disease 2019 (COVID-19) and other viral diseases is still under debate, since they also have potent pro-inflammatory effects.^3–5^ Moreover, contradictory findings were observed regarding the importance of IFN in the immunopathology of COVID-19. Increased IFN production was found in patients with reduced pulmonary function and the poorest prognosis^6^ while some studies reported reduced IFN responses in COVID-19 patients with severe disease.^7^ The interaction between IFN and the vascular system may be central in the progression of severe COVID-19 and associated long-term sequelae. Whether IFNα or IFNλ3 has direct effects on vascular cells in the context of SARS-CoV-2 infection remains unclear.

The entry receptor for SARS-CoV-2 is the angiotensin converting enzyme 2 (ACE2) expressed in several cell types, including epithelial, immune, and vascular cells. The spike protein located on the viral envelope binds to ACE2 and is proteolytically cleaved by the transmembrane protease serine protease 2 (TMPRSS2), resulting in virus entry.^8^ The ectodomain of ACE2 is shed from the cell surface by the disintegrin and metalloproteinase domain 17 (ADAM17), increasing the soluble form of ACE2 in the circulation and has been associated with increased cardiovascular comorbidities observed in COVID-19.^9^ Of importance, increased ADAM17 activity is associated with cardiometabolic diseases, including hypertension, atherosclerosis and diabetes mellitus.^10^

Despite the tropism of SARS-CoV-2 for the lungs, causing interstitial pneumonitis, interactions with cells in the cardiovascular system have been documented. SARS-CoV-2 was found in endothelial and cardiac cells from COVID-19 patients and in vessels from human organoids.^11–13^ In previous studies, we demonstrated that SARS-CoV-2, as well as SARS-CoV-2 spike protein S1 subunit (SP1), induces inflammation in endothelial cells (ECs) in part through ACE2.^14^ These findings suggest that the endothelium may be a key player in systemic and local inflammation and may contribute to vascular injury in COVID-19.^15^

Endothelial cells express innate immune receptors and respond to circulating inflammatory mediators. Plasma from severe COVID-19 patients exhibits elevated levels of IFN-β that can further contribute to chronic inflammation.^16^ This may be especially important in the context of hypertension and cardiovascular diseases, which are increasingly recognized as sequelae of COVID-19.^17–19^ Despite the growing understanding of the effects of IFNs in epithelial cells in viral infections, it is still unknown whether IFNs promote vascular inflammation in COVID-19. To address this, we investigated whether IFN responses mediated by SP1 of SARS-CoV-2 induce activation of pro-inflammatory pathways in human ECs. We also explored the role of these processes in vascular function in the context of hypertension. We focused on ISG15, since this ISG is crucial in immune/anti-viral responses and also plays an important role in hypertension-associated vascular injury, as we reported.^20^

## 2. Methods

### 2.1 Cell culture

#### 2.1.1 Human endothelial cells

All cells and culture media were acquired from Promocell: Human pulmonary artery ECs (HPEC) (C-12241) and human aortic ECs (HAEC) (C-12271) were kept in Endothelial Cell Growth Medium ready to use (C-22010). Human dermal microvascular ECs (HMEC) (C-12210) and human dermal lymphatic EC (HLEC) (C12216) were cultured in Endothelial Cell Growth Medium MV2 (C-22022). Culture media were supplemented with Penicillin/Streptomycin (50 µg/mL) (Invitrogen). For this study, at least three vials of different EC populations were acquired. According to the information provided by the company, cells were isolated from different parts of the body (abdomen, thorax, labia, etc.) from adults. Experiments were performed until Passage 6. All cells were free of mycoplasma contamination, as certified by the company and routinely tested in our laboratory. For the experiments, medium was changed to DMEM (Invitrogen, Thermo-Fisher-Gibco, Ottawa, Ontario, Canada) supplemented with 0.5% of foetal bovine serum (FBS) (Thermo-Fisher-Gibco, Ottawa, Ontario, Canada, cat A5670801) and penicillin/Streptomycin 2 h before stimulation.

#### 2.1.2 Cell lines deficient in STAT1 and IFNLRI

Two A549-derived cell lines, A495-Stat1KO and A495-IFNLR1KO cells, were generated by CRISPR-Cas9 ‘nickase’ DNA plasmid co-transfection. Plasmid pSpCas9n(BB)-2A-Puro (PX462) V2.0 was gifted by Prof Feng Zhang (Broad Institute of Massachusetts Institute of Technology [MIT] and Harvard, Cambridge, MA) ^21^ Plasmids for CRISPR-Cas9 genome editing (SpCas9) used as guide sequences were: STAT1: TCATGACCTCCTGTCACAGC and TCATTGGCAGCGTGCTCCCT.

IFNLR1: ACTTCAGCGTGTACCTGACA and GGGAGAGCAGCGTCACATTC.

A495 cells were co-transfected with each nickase plasmid using lipofectamine 2000 (Thermo Fisher cat 11668019), followed by puromycin (Thermo Fisher, cat. A1113803) (1 μg/mL) selection and single cell cloning prior to further characterization of gene knockout for STAT1 and IFNLR1. After transfection, A495 cells were kept in DMEM/10% FBS.

Cells were stimulated with IFNα (100 IU/mL, cat. 11105–1), IFNλ3 (100 ng/mL, cat. 5259-IL-025), or Poly(I:C) (100 ng/mL, cat. 4287/10) as a positive control (all from R&D Systems), all diluted in phosphate-buffered saline (PBS). In another set of experiments, cells were stimulated with recombinant SARS-CoV-2 spike protein, S1-subunit (SP1) (1 µg/mL, RayBiotech, cat. 230-01101-100) or recombinant SARS-CoV-2 spike protein, S2-subunit (SP2) (1 µg/mL, RayBiotech, cat. 230-01103-100) using PBS as vehicle. Recombinant proteins were undetected for endotoxin levels, as certified by the company, and also tested in-house using the Pierce Rapid Gel Clot Endotoxin Assay Kit (cat A43879). The concentrations used are based on dose–response experiments (see Supplementary material online, *Figure S2*) and confirmed in other studies.^22,23^ The S1 subunit contains the receptor-binding domain, which facilitates binding of the virus to ACE2 of the host cell. The S2 subunit is responsible for triggering the virus-host membrane fusion.^24^

Some experiments were performed in the presence of an ACE2 inhibitor (MLN-4760, 5 nmol/L, Sigma-Aldrich, cat. 5306160001), ADAM17 inhibitors (marimastat, 3.8 nmol/L, Sigma Aldrich, cat. M2699 and TAPI-0, 100 nmol/L, Tocris—cat. 5523). Marimastat and TAPI-0 are both metalloproteinase inhibitors. Marimastat is a broad-spectrum inhibitor that inhibits ADAM10 and ADAM17, whereas TAPI-0 has greater specificity to ADAM17. TMPRSS2 inhibitor (camostat mesylate, 50 µmol/L, Sigma-Aldrich, cat. SML0057) and STAT1 inhibitor (Fludarabine phosphate, 1 μmol/L, Sigma-Aldrich, cat. F98130), all diluted in DMSO. The maximum final DMSO added to cells was 0.25% (v/v). A similar % of DMSO was added to the vehicle control groups.

#### 2.1.3 siRNA transfection experiments

Human MEC were plated (80%–90% confluent) and cultured for 24 h in growth medium supplemented with antibiotics. Cells were incubated with 20 nmol/L of IFNLR1 (IL28RA) siRNA (Silencer Select, Thermo Fischer, catalogue number 4392420, siRNA ID s223390, lot number A50204V1) complexed with Lipofectamine RNAiMAX (Thermo Fischer Scientific, cat number 13778075) as transfection reagent in DMEM without serum and antibiotics for 6 h. A sequence not homologous to any gene in the vertebrate transcriptome was used as control siRNA (Negative Control No. 1 siRNA, Ambion, Catalogue number 4390844, lot number A502GNGX). After transfection, medium was replaced with growth medium, and experiments were conducted 48 h after transfection.

### 2.2 Nitric oxide (NO) production

Production of NO was determined using the NO fluorescent probe diacetate 4-amino-5-methylamino-2′,7′-difluorofluorescein diacetate (DAF-FM; Life Technologies, Molecular Probes), according to manufacturer instructions. Briefly, MEC were loaded with DAF-FM diacetate (final concentration 5 µmol/L, 30 min) in serum-free media, kept in the dark, and maintained at 37°C. Cells were washed to remove excess probe. Fresh medium was added and incubated for an additional 10 min to allow complete de-esterification of the intracellular diacetates. Cells were stimulated with IFNα (100 IU/mL) or IFNλ3 (100 ng/mL) for 30 min, washed with PBS and harvested with 0.025% trypsin. Trypsin was inactivated with soybean trypsin inhibitor (0.025%) in PBS (1:1). After washing, the pellet was transferred to a black 96-well microplate (BD Falcon). The DAF-FM fluorescence was assessed with a spectrofluorometer at excitation/emission wavelengths of 495/515 nm. Fluorescence intensity was normalized to the protein concentration and expressed as fluorescence emission/µg of protein.

### 2.3 Immunoblotting

Total protein was extracted from cells using lysis buffer (Tris 50 mmol/L, pH 8.0, NaCl 150 mmol/L, Triton X-100 1%, SDS 0.1%) supplemented with phenylmethylsulfonyl fluoride (PMSF) 1 mmol/L, pepstatin A 1 µg/mL, leupeptin 1 µg/mL, aprotinin 1 µg/mL (Sigma-Aldrich), sodium fluoride 10 mmol/L (AnalaR Normapur; VWR International), and sodium orthovanadate 1 mmol/L (Alfa Aesar). Total protein lysate was sonicated (10 kHz, 5 s, 4°C), cleared by centrifugation at 5000*g* for 5 min, and the pellet was discarded. Protein concentration was determined using the DC protein assay kit (Pierce—ThermoFisher Scientific).

Total protein (20 µg) was separated by electrophoresis on a gradient polyacrylamide gel (NuPAGE 4 to 12%, Bis-Tris, 1.0 mm, Invitrogen) and transferred onto a nitrocellulose membrane (Thermo Scientific). Non-specific binding sites were blocked with 5% non-fatty dry milk solubilized in Tris-buffered saline solution with Tween 0.01% for 30 min at room temperature. Membranes were incubated overnight at 4°C with the following primary specific antibodies: ACE2 (mouse, cat. 66699-1-Ig), total-STAT2 (mouse, cat. 66485-1-Ig), PAI-1 (mouse, cat. 66261-1-Ig), PCNA (rabbit, cat. 10205-1-Ig) all from Proteintech, dilution 1:1000; ADAM17 (TACE, rabbit, D22H4, cat.6978), phospho-STAT2 (Tyr690) (rabbit, D3P2P, cat 88410), phospho-STAT1 (Tyr701) (rabbit, D4A7, cat. 7649S), total-STAT1 (mouse, 9H2, cat. 9176S), phospho-P38 MAPK (Thr180/Tyr182) (rabbit, cat. 9211S), total-P38 (rabbit, cat. 9212S), phospho-eNOS (Ser1177) (rabbit, cat. 9572), phospho-eNOS (Thr495) (rabbit, cat. 9545) from Cell Signalling, dilution 1:1000; Total eNOS (mouse, Santa Cruz, dilution 1:500); TMPRSS2 (rabbit, EPR3861, Abcam, cat. 92323, 1:1000); β-actin (mouse, Sigma-Aldrich, 1:5000).

Membranes were washed with TBS-tween and incubated with secondary fluorescent labelled antibodies, either goat anti-Rabbit IgG (H + L)-Alexa Fluor Plus 800 (cat. A32735) or goat anti-Mouse IgG (H + L)-Alexa Fluor 680 (A21057) (Thermo Fisher, dilution 1:5000) for 1 h, at room temperature in the dark. Fluorescence intensity was visualized and acquired by an infrared laser scanner (Odyssey Clx, LICOR, Cambridge, UK, or Sapphire FL imager, Azure Biosystems, CA, USA). Images were quantified using the software Image Studio Lite free version (LICOR, Cambridge, UK). Protein expression levels were normalized to loading controls.

### 2.4 Flow cytometry

#### 2.4.1 Intracellular staining

Semi-confluent MEC and LEC cultured in 12-well plates were rendered quiescent for 2 h in DMEM/FBS 0.5% and stimulated with SP1 (1 μg/mL). After 24 h, cells were detached from the plate using the enzyme-free reagent ReleSR (StemCell technologies, cat. 100-0483) for 10 min. Cells were washed with PBS/FBS 5% and resuspended in PBS/FBS 1%. Next, cell suspensions were treated with Zombie NIR Fixable Viability Kit (BioLegend, Inc. cat. 423105, dilution 1:1000) for 15 min, at room temperature, in the dark, followed by washing twice with PBS. Next, cells were permeabilized using the Foxp3/Transcription Factor Staining Buffer Set (eBioscience, cat. 00-5523-00), according to manufacturer instructions. Briefly, cells were resuspended in fixation/permeabilization buffer and incubated at room temperature for 1 h. After, cells were resuspended in permeabilization buffer with PE-anti-human IL-28RA (IFNLR1, clone MHLICR2a, 1:400, Biolegend cat. 337803) or isotype control (PE Mouse IgG2a, κ, 1:400, Biolegend, cat. 400213) and incubated for 30 min at room temperature, in the dark. Cells were washed twice by centrifugation and resuspended in PBS/1% FBS. Data acquisitions were performed in the FACS Canto II flow cytometer (BD Biosciences) and analysed using FlowJo software (TreeStar, Ashland).

#### 2.4.2 Proliferation assay

The cell tracking dye carboxyfluorescein succinimidyl ester (CellTrace CFSE; cat. C34554, Thermo Fisher) was used in the proliferation assay. Following each cell division, the fluorescein-tagged cellular molecules are split evenly in two daughter cells, and cell proliferation assessed by measuring CFSE fluorescence. Briefly, MEC were incubated with 5 µmol/L CFSE in PBS containing 1% (v/v) FBS for 30 min at 37°C. After incubation, CFSE-labelled cells were washed twice using PBS, and then plated at ∼30% confluence in six-well plate using EC Growth Medium MV2. After 24 h, cells were detached from the plate, resuspend in 300 μL of PBS/1% FBS and CFSE fluorescence was detected with an FITC channel, using Flow Cytometry (FACS Canto II, BD Biosciences). Data were analysed using the FlowJo software (TreeStar, Ashland).

### 2.5 Real-time reverse-transcription polymerase chain reaction

Total RNA was isolated using the QIAzol Lysis Reagent (Qiagen) according to the manufacturer’s instructions and diluted in nuclease-free H_2_O (Ambion/Life Technologies). cDNA was generated from total RNA using the High-Capacity cDNA Reverse Transcription Kits (Applied Biosystems). Real-time polymerase chain reaction (RT–PCR) assays were performed with the Applied Biosystems QuantStudio 12K Flex RT–PCR system or Bio-Rad CFX96 Touch Real-Time PCR qPCR System using Power SyBr Green Master Mix (Applied Biosystems). Specific human primers (see Supplementary material online, *Table S1*) were acquired from Eurofins Genomics (Glasgow, UK) or from Invitrogen—Life Technologies (CA). Relative gene expression was calculated by the 2^−ΔΔCt^ method.

### 2.6 Endothelial cell production of pro-inflammatory mediators

The following pro-inflammatory biomarkers were analysed in cell supernatant using human DuoSet ELISA kits acquired from R&D Systems: IL-1 beta/IL-1F2 (cat. DY201), IL-6 (cat. DY206), CCL2/MCP-1 (cat. DY279), IL-8/CXCL8 (cat. DY208).

The following soluble markers were analysed in the cell supernatant using human MILLIPLEX Multiplex Assays acquired from Merck Life Science UK: TNFα (cat. HCYTA-60K-04); soluble VCAM-1 (sVCAM-1) and sICAM-1 (cat. HAGP1MAG-12K-02); Endothelin-1 (ET-1) and angiopoietin-2 (Angpt2) (cat. HCVD2MAG-67K-02).

### 2.7 Cell migration

MEC migration was assessed using the scratch-wound assay, a two-dimensional (2D) *in vitro* technique. Confluent MEC cultured in 12-well plates were rendered quiescent for 2 h in DMEM with 0.5% FBS. Next, a sterile pipette tip was used to scratch the cell monolayer, making a straight ‘wound’ with equal width. The medium was then replaced with fresh DMEM 0.5% FBS to remove floating cells and reduce the effects of cell proliferation. Three images were acquired from each well at 0 h and after 24 h using a 10× objective (ECHO Rebel, Echo Biocompany). Images were collected and analysed using ImageJ software (National Institutes of Health, Bethesda, MD).

### 2.8 Mouse studies

All experimental protocols were performed in accordance with ARRIVE Guidelines, the United Kingdom Animals Scientific Procedures Act 1986 (Licence No. 70/9021). Studies were conducted according to the University of Glasgow Animal Welfare and Ethics Review Board. Studies conducted at the Universidad Autónoma de Madrid (Spain) were in accordance with the Spanish Policy for Animal Protection RD53/2013, which meets the European Union Directive 2010/63/UE on the protection of animals used for experimental and other scientific purposes. Animal studies conducted at the Research Institute of McGill University Health Centre (RIMUHC), Montreal, Canada, were in accordance with the Canadian Council on Animal Care (CCAC) regulations. The animal protocol was approved by the Animal Care Committee of the RIMUHC, licence number MUHC-10018. For this study, we used male mice from the following strains: LinA3 (TTRhRen) and control WT mice (FVB background, 24–28 weeks old), and ISG15−/− mice and control WT (ISG+/+) littermate controls (C57BL/6J background), which were kindly provided by Dr Klaus-Peter Knobeloch (University of Freiburg, Germany). These mice were bred at the conventional Animal Care Facility of the Faculty of Medicine (UAM). Animal care and experimental procedures were approved by the Ethical Committee of Research of the Universidad Autónoma de Madrid and Dirección General de Medio Ambiente, Comunidad de Madrid, Spain (PROEX 345/14 and 183.2/20).

Animals were housed under 12 h light/dark cycles at ambient temperature and were maintained on a normal mouse diet. Mice were euthanized by overdose of anaesthesia (5% isoflurane in 1.0 L/min O_2_) until respiration ceased, followed by cervical dislocation. Vascular function was assessed in small resistance arteries (which contribute to blood pressure elevation) from LinA3 (TTRhRen) and WT mice. LinA3 mice express human prorenin under the control of the transthyretin promoter and are hypertensive, as we previously described.^25^ These mice have been fully characterized and gradually develop hypertension with ageing and recapitulate human essential hypertension.^26,27^ Vascular studies were also assessed in ISG15−/− mice and their control counterparts.

Systolic blood pressure (SBP) was measured by tail-cuff plethysmography (BP 2000 Blood Pressure Analysis System, Visitech, Science Products GmbH, Germany). Mice were immobilized on a warmed platform at 37°C. Blood pressure was measured in conscious mice. The first five recordings were discarded, and the average of the 10 successive measurements was taken as the final blood pressure reading.

### 2.9 Assessment of vascular function

Vascular function was investigated by myography in mesenteric resistance arteries from LinA3, ISG15−/− and WT mice. First- and second-order vessels were cut into 2 mm ring segments and mounted on a wire myograph (Danish Myo Technology). Myograph chambers were filled with 5 mL of Krebs–Henseleit physiological solution [(in mmol/L): NaCl 130, NaHCO_3_ 14.9, KCl 4.7, KH_2_PO_4_ 1.18, MgSO_4_•7H_2_O 1.17, glucose 5.5, CaCl_2_•2H_2_O 1.56, and EDTA 0.026] (all from Sigma-Aldrich) and continuously gassed with a mixture of 95% O_2_ and 5% CO_2_ at a temperature of 37°C. After 30 min of stabilization, contractile responses were assessed by adding KCl 120 mmol/L (Sigma-Aldrich) to the organ baths. Endothelial integrity was verified by relaxation induced by acetylcholine (Ach; 10^−6^ mol/L) (Sigma-Aldrich) in vessels pre-contracted with U46619 (10^−7^ mol/L) (Sigma-Aldrich).

Vessels were treated as follows: 1) vehicle; IFNα (100 IU/mL); IFNλ3 (100 ng/mL) for 1 h; SP1 (1 μg/mL, 2 h) or recombinant SARS-CoV-2 Spike (R&D systems, Cat: 10549-CV, 1 μg/mL for 2 h) and precontracted with U46619 (10^−7^ mol/L). Endothelium-dependent relaxation was assessed by concentration-responses to Ach (10^−9^ mol/L to 10^−4^ mol/L); 2) Vessels were washed and incubated with similar concentrations of IFNα, IFNλ3, SP1, or vehicle for 30 min. Contraction was assessed by cumulative concentration–response curves to U46619 (10^−9^ mol/L to 3 × 10^−4^ mol/L). 3) Vessels were washed and incubated with similar concentrations of IFNα, IFNλ3, SP1 or vehicle for 30 min. Endothelium-independent relaxation was assessed by cumulative concentration curves to sodium nitroprusside (SNP) (10^−10^ mol/L to 3 × 10^−4^ mol/L) in vessels precontracted with U46619 (10^−7^ mol/L). All stimuli were kept during the curves.

### 2.10 Statistical analysis

Data were analysed using GraphPad Prism 10. Significance was assumed if *P* < 0.05. Data are presented as mean ± SEM. Two-tailed unpaired Student's *t*-test was used when differences between two groups were analysed. Analysis of variance (ANOVA) and Dunnett's multiple comparisons test were used to evaluate the statistical significance of differences between three or more groups. For vascular function, concentration–response data were analysed by determining EC50 and maximal response (*E*max) values from experimental data fitted to a four-parameter logistic function against the null hypothesis. Statistical significance was determined using two-way ANOVA followed by Bonferroni’s post-test for multiple comparisons. Statistical values are shown in the figures.

## 3. Results

### 3.1 Spike protein S1 increases expression of interferon-stimulated genes in endothelial cells

**Figure 1:**
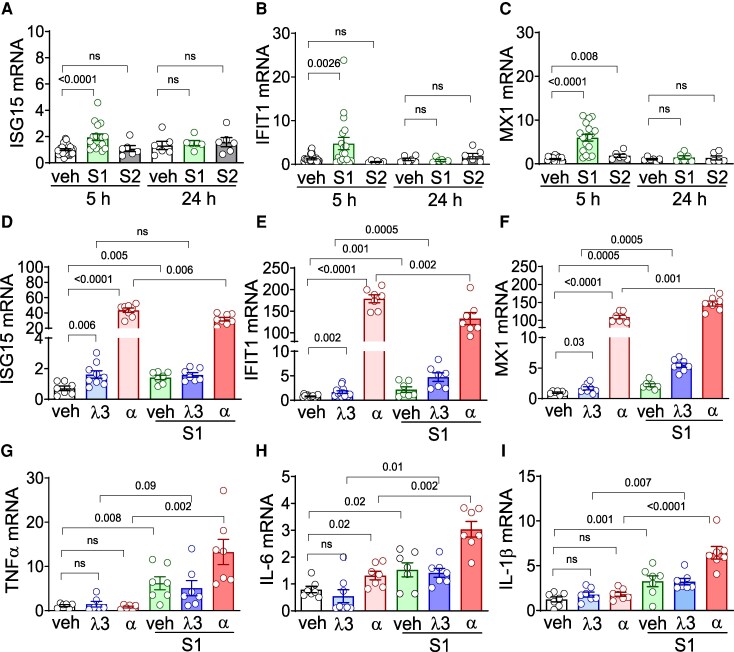
Spike protein S1 of the SARS-CoV-2 (SP1) induces expression of ISGs and enhances inflammatory response induced by IFNs in human microvascular endothelial cells. (*A–C*) Microvascular endothelial cells (MEC) were stimulated with 1 µg/mL of spike protein 1 (S1) or spike protein 2 (S2) for 5 and 24 h. (*D–I*) MEC were treated with IFNλ3 (100 ng/mL) or IFNα (100 IU/mL) for 4 h and then stimulated with S1 (1 µg/mL) for additional 5 h. Gene expression for (*D*) ISG15, (*E*) IFIT1, (*F*) MX1, (*G*) TNFα, (*H*) IL-6, and (*I*) IL-1β was investigated by real time PCR and normalized by GAPDH (*n* = 7–8). One-way ANOVA followed by Dunnett’s multiple comparisons test were used for statistical analysis. Statistically significant *P*-values are shown. NS—Not Significant.

We first investigated the expression of proteins required for SARS-CoV-2 interaction with host cells. Endothelial cells: aortic (AEC), pulmonary (PEC), microvascular (MEC), and lymphatic (LEC) express ACE2, ADAM17, and TMPRSS2 (see Supplementary material online, *Figure S1*). MEC treated with SP1 exhibited increased mRNA expression for ISGs (ISG15, IFIT1, and MX1) (see Supplementary material online, *Figure S2A*–*C*) and IFNα and IFNλ (see Supplementary material online, *Figure S2D*–*G*) in a concentration-dependent manner. Endothelial cells from different vascular/lymphatic beds treated with SP1 exhibited increased mRNA expression of ISG15, IFIT1, and MX1 (*Figure 1A–C* and Supplementary material online, *Figure S3*). Minor effects were observed on ISG15 expression in AEC and PEC stimulated by SP2 after 24 h stimulation (see Supplementary material online, *Figure S3D* and *G*). All endothelial cell populations expressed ISGs after activation with the positive control poly(I:C), indicating their immune-reactivity (see Supplementary material online, *Figure S4*).

**Figure 2:**
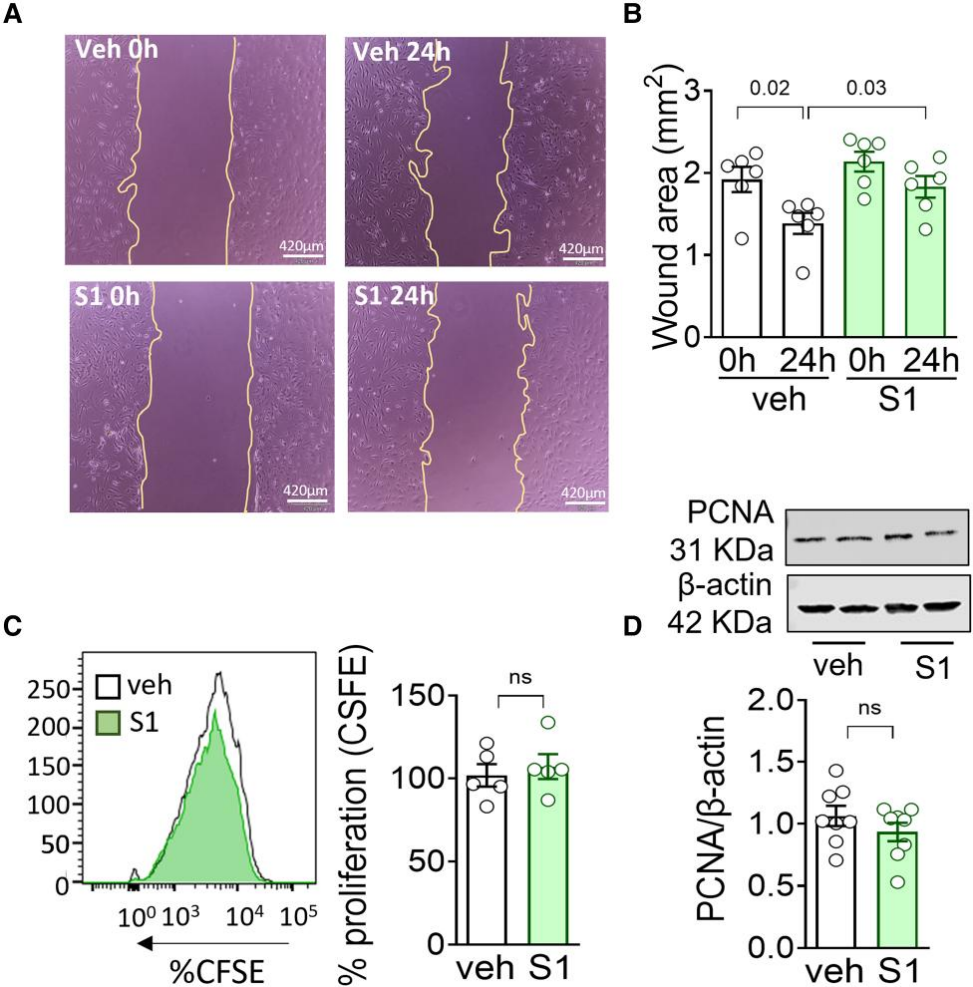
Spike protein S1 of the SARS-CoV-2 (SP1) reduces microvascular endothelial cells migration. Microvascular endothelial cells (MEC) were treated with spike 1 protein (S1) (1 µg/mL) for 24 h. (*A–B*) Cell migration was assessed by Scratch-wound assay. Pictures were acquired at time zero and 24 h. (*C*) MEC were incubated with 5 µM CFSE and stimulated with S1 (1 µg/mL). Fluorescence intensity was assessed by flow cytometry after 24 h (*n* = 6). (*D*) Protein expression of PCNA was investigated by western-blotting and normalized by β-actin. Statistical analysis was conducted using a two-tailed unpaired Student’s *t*-test. Statistically significant *P*-values are shown. NS—Not Significant.

IFNλ3 and IFNα are important mediators of innate immunity in viral infection, and levels are increased in plasma from COVID-19 patients.^28^ When MECs were exposed to IFNs, mRNA expression of ISG15, IFIT1, MX1 was increased (*Figure 1D–F*). SP1 amplified these effects in an IFN-specific manner: SP1 increased IFNλ3-induced expression of IFIT1and MX1 (*Figures 1E and 2F*) and increased IFNα and IFNλ3 effects on TNFα, IL-6, and IL1β (*Figure 1G–I*). Of importance, SP1 did not upregulate effects of IFNs in LEC (see Supplementary material online, *Figure S5A*). LEC, AEC, and PEC exhibited increased ISGs only by IFNα stimulation (see Supplementary material online, *Figure S5A*–*C*). Differential effects of IFNλ3 on different EC are not due to receptor expression, since similar expression of the IFNλ3 receptor IFNLR1 was observed in MEC and LEC, which was not changed by SP1 stimulation (see Supplementary material online, *Figure S6*).

Next, we investigated two functional parameters of ECs: migration and proliferation, both of which are important processes in endothelial dysfunction, vascular inflammation, and remodelling. Following SP1 stimulation for 24 h, migration was reduced in MECs compared with their control counterpart (*Figure 2A and B*), as observed by no changes in the wound area. SP1 had no significant effect on cell proliferation as observed by CSFE flow cytometry assay and PCNA protein expression (*Figure 2C and D*).

### 3.2 ADAM17 inhibitors reduce expression of ISGs induced by spike protein 1.

**Figure 3:**
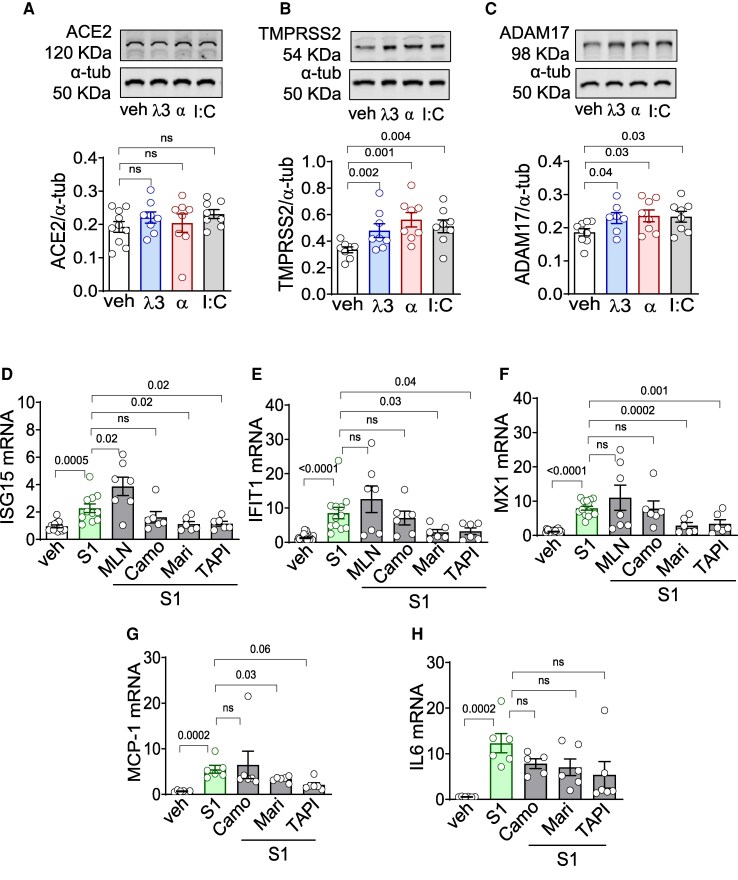
ADAM17 inhibitor reduces expression of ISGs induced by spike protein 1 in microvascular endothelial cells. (*A–C*) MEC were treated with IFNλ3 (100 ng/mL), IFNα (100 IU/mL) or poli(I:C) (100 ng/mL) (*n* = 8). Protein expression for (*A*) ACE2, (*B*) TMPRSS2 and (*C*) ADAM17 was investigated after 24 h stimulation and normalized by α-tubulin. (*D–H*) MEC were pre-treated with MLN-4760 (ACE2 inhibitor), camostat mesylate (TMPRSS2 inhibitor), marimastat and TAPI-0 (ADAM17 inhibitors) for 30 min and stimulated with spike protein 1 (S1). Gene expression for (*D*) ISG15, (*E*) IFIT1, (*F*) MX1, (*G*) MCP1 and (*H*) IL-6 was investigated after 5 h stimulation and normalized by GAPDH (*n* = 7–12). Group analyses were determined by one-way ANOVA followed by Dunnett’s multiple comparisons test. Statistically significant *P*-values are shown. NS—Not Significant.

ACE2, TMPRSS2, and ADAM17, proteins involved in SARS-CoV-2 entry into cells, were detected in ECs. In MEC, IFNλ3 and IFNα increased protein expression of TMPRSS2 and ADAM17, but not ACE2 (*Figure 3A–C*). IFNλ3 also increased protein expression of TMPRSS2 in LEC and PEC (see Supplementary material online, *Figure S7A* and *C*), whereas IFNα increased expression ACE2 in PEC and TRPMRSS2 in all EC populations (see Supplementary material online, *Figure S7*).

To investigate the role of ADAM17, TMPRSS2, and ACE2 in SP1-induced ISG responses in MEC, cells were exposed to pharmacologic inhibitors of these proteins. Only ADAM17 inhibition with marimastat or TAPI-0 reduced mRNA expression of SP1-stimulated ISG15, MX1 and IFIT1 (*Figure 3D–F*) as well as MCP-1 (*Figure 3G and H*).

### 3.3 STAT activation and inflammatory responses in IFNλ3 and IFNα -stimulated microvascular endothelial cells

**Figure 4:**
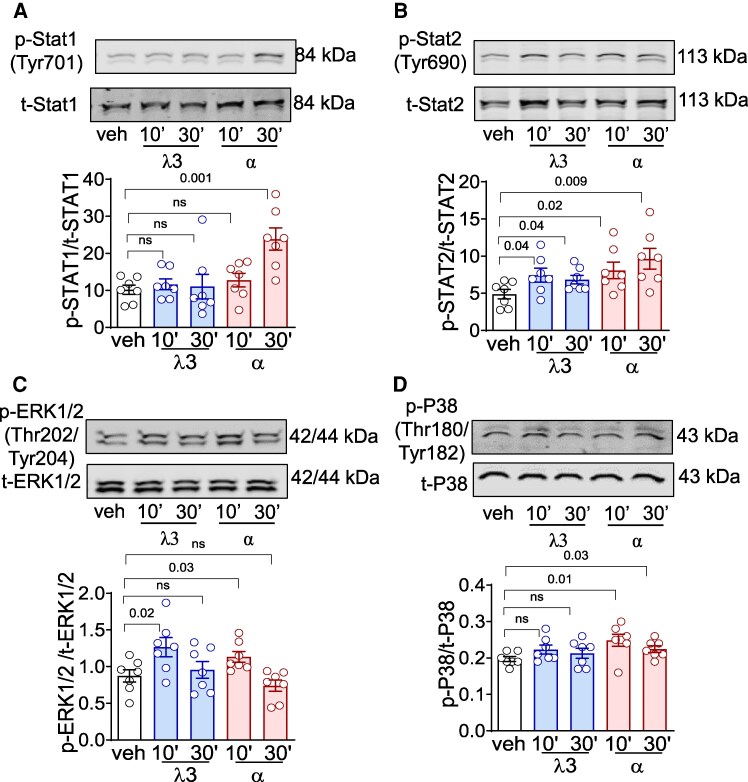
IFNλ3 and IFNα activate STAT and MAPK signalling pathways in microvascular endothelial cells. MEC were treated with IFNλ3 (100 ng/mL) or IFNα (100 IU/mL) for 10 and 30 min. Protein expression for (*A*) p-STAT1(Tyr701), (*B*) p-STAT2 (Tyr690), (*C*) p-ERK1/2 (Thr202/Tyr204) and (*D*) p-P38 (Thr180/182) was investigated by western blotting and normalized by total proteins (*n* = 7). Group analyses were determined by One-way ANOVA followed by Dunnett’s multiple comparisons test. Statistically significant *P*-values are shown. NS—Not Significant.

IFNλ3 and IFNα interact with specific receptors in the plasma membrane of the target cell and induce intracellular signalling pathways dependent on the activation of the transcription factors STAT1 and STAT2 and MAP kinases, ERK1/2, and p38MAPK.^2^ Acute phosphorylation of STAT1, STAT2, ERK1/2, and P38MAPK was observed in MEC stimulated with IFNα (*Figure 4A–D*), whereas IFNλ3 activation induced phosphorylation of STAT2 and ERK1/2. Both IFNs induced phosphorylation of STAT1 and STAT2 at 24 h (see Supplementary material online, *Figure S8*). However, in LEC, this activation pathway was observed only for IFNα stimulation (see Supplementary material online, *Figure S9*).

**Figure 5:**
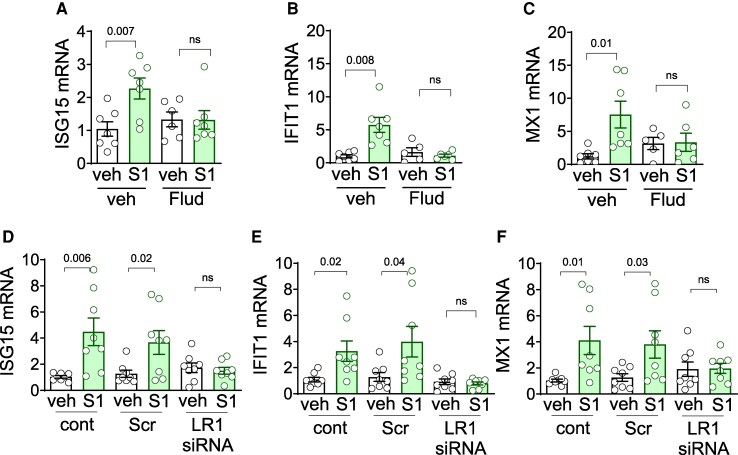
Inhibition of STAT1 and IFNLR1 reduces ISGs gene expression induced spike protein S1. (*A–C*) Microvascular endothelial cells (MEC) were pre-treated with fludarabine (STAT1 inhibitor, 1 μmol/L) for 30 min and stimulated with spike protein 1 (S1) (1 μg/mL). (*D–F*) MEC were treated with IFNLR1 silencing RNA and stimulated with S1 (1 μg/mL). Gene expression for ISG15, IFIT1, and MX1 was investigated after 5 h stimulation and normalized by GAPDH (*n* = 7–9). Group analyses were determined by One-way ANOVA followed by Dunnett’s multiple comparisons test. Statistically significant *P*-values are shown. NS—Not Significant.

To further explore the role of STAT1 and IFNLR1 in SP1-induced regulation of ISGs, we used two approaches to downregulate the system, including pharmacological inhibitors and gene-silencing. Cells were treated with the STAT1 inhibitor fludarabine and silencer RNA for IFNLR1 (see Supplementary material online, *Figure S10*). In MEC exposed to fludarabine (*Figure 5A–C*) or IFNLR1 siRNA (*Figure 5D–F*), SP1 failed to increase mRNA expression of ISGs. These effects were also confirmed in STAT1-deficient and IFNLR1-deficient A495 cells (see Supplementary material online, *Figure S11*).

**Figure 6:**
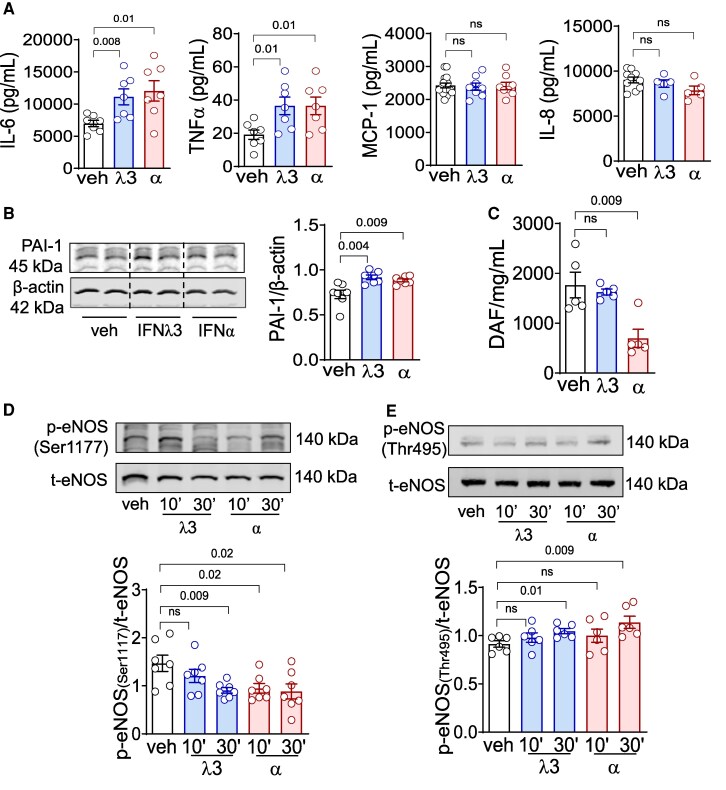
IFNλ3 and IFNα induce a pro-inflammatory phenotype and eNOS inhibition in microvascular endothelial cells. Microvascular endothelial cells (MEC) were treated with IFNλ3 (100 ng/mL) or IFNα (100 IU/mL). The production of (*A*) IL-6 (*n* = 7), TNFα (*n* = 7), MCP-1 (*n* = 8–12), IL-8 (*n* = 5), and (*B*) protein expression for PAI-1 (*n* = 7) were investigated after 24 h stimulation. Protein expression was normalized by α-tubulin. (*C*) Nitric oxide (NO) production was accessed by DAF fluorescent assay after 30 min stimulation and normalized by total protein expression (*n* = 5). (*D–E*) Phosphorylation of eNOS (Ser1177) (activation motif) and eNOS (Thr495) (inhibitory motif) was investigated after 10 and 30 min and normalized by total-eNOS expression (*n* = 6–7). Group analyses were determined by One-way ANOVA followed by Dunnett’s multiple comparisons test. Individual effects were determined by a two-tailed unpaired Student’s *t*-test. Statistically significant *P*-values are shown. NS—Not Significant.

A final consequence of activation of pro-inflammatory signalling pathways is the production of inflammatory mediators and adhesion molecules. Production of IL-6, TNFα, and protein expression of PAI-1, but not MCP-1 and IL-8, was increased in MEC stimulated with IFNλ3 and IFNα (*Figure 6A and B*).

One of the most important functions of ECs is activation of eNOS to produce the vasodilator NO. NO production was reduced by IFNα stimulation (*Figure 6C*). IFNα and IFNλ3 blunted activation of eNOS as indicated by reduced phosphorylation of the eNOS activation motif Ser1177 and increased phosphorylation at the inhibitor motif Thr495 (*Figure 6D and E*). IFNs had no effect on the production of soluble VCAM-1 (sVCAM-1), sICAM-1, endothelin-1 (ET-1), and angiopoietin-2 (Angpt2) (see Supplementary material online, *Figure S12*). Only IFNα increased production of IL-6 in AEC and PEC (see Supplementary material online, *Figure S13*).

### 3.4 Vascular dysfunction induced by IFNλ3, IFNα and spike protein involves ISG15

**Figure 7:**
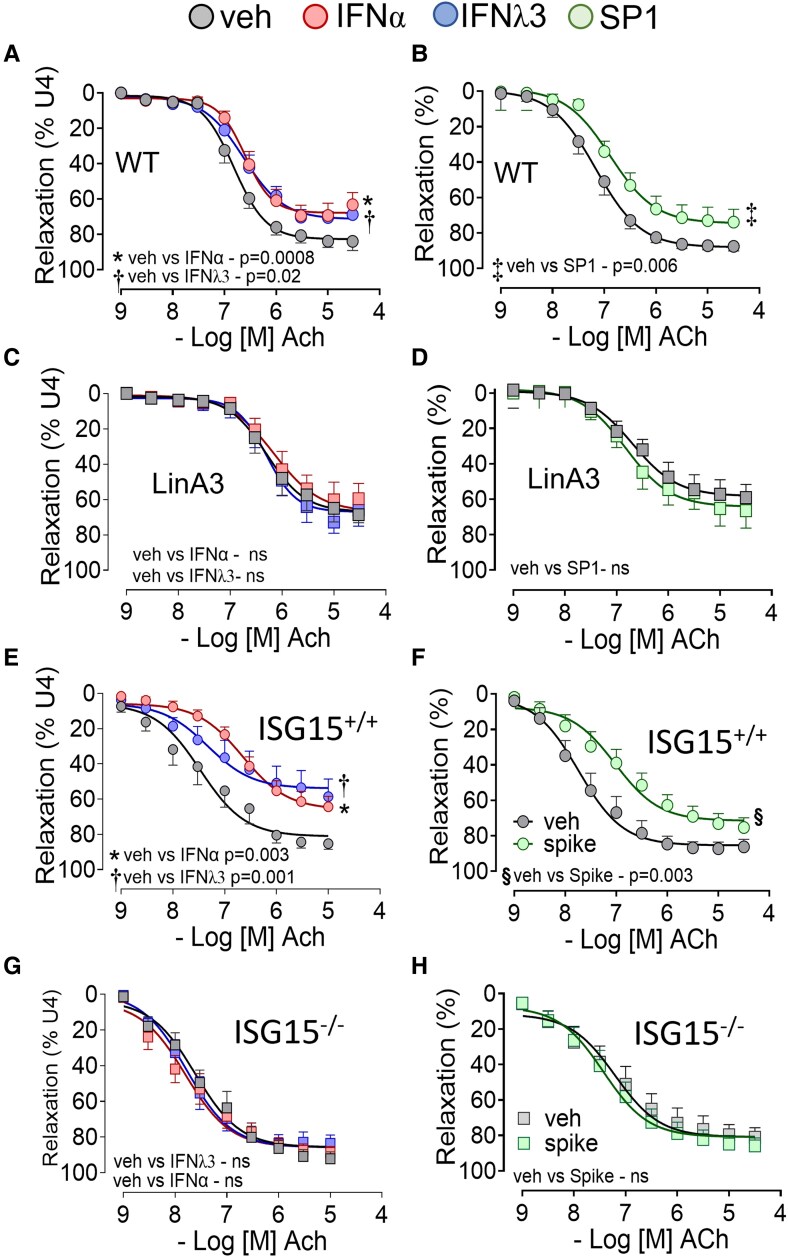
IFNλ3, IFNα and spike protein reduce endothelium-dependent relaxation by ISG15 dependent mechanisms. (*A–D*) Small mesenteric vessels were isolated from (*A*, *B*) wildtype (WT) (circles, *n* = 8) and (*C*, *D*) hypertensive LinA3 (squares, *n* = 6) mice. Vessels were mounted on wire myographs and treated with IFNλ3 (100 ng/mL, blue), IFNα (100 IU/mL, red) for 1 h or SP1 (1μg/mL, green) for 2 h, followed by concentration-response curves to acetylcholine (Ach)-relaxation. (*E–H*) Small mesenteric vessels were isolated from (*E*, *F*) ISG+/+ (circles, *n* = 8) or (*G*, *H*) ISG−/− (squares, *n* = 6) mice. Vessels were mounted on wire myographs and treated with IFNλ3 (100 ng/mL, blue), IFNα (100 IU/mL, red) for 1 h or spike protein (1 μg/mL, greeen) for 2 h, followed by concentration-response curves to acetylcholine (Ach)-relaxation. Ach curves are expressed in % of relaxation compared to pre-constriction induced by U44619. Vascular function, maximal response (*E*max) values from experimental data were fitted to a four-parameter logistic function against the null hypothesis and two-way ANOVA followed by Bonferroni’s post-test for multiple comparisons. Statistically significant *P*-values are shown. *veh vs. IFNα, ^†^veh vs. IFNλ3, ^‡^veh vs. SP1, ^§^veh vs. spike protein.NS—Not Significant.

To determine the effects of IFNs in intact vessels, we assessed vascular function in isolated small arteries (*Figure 7*). To investigate the effects of hypertension, we used LinA3 mice, which is a model of renin-dependent hypertension. These mice have been extensively characterized and recapitulate the development of hypertension in humans.^25–27^ LinA3 mice had significantly elevated systolic blood pressure (160 ± 24.7 mmHg vs. WT, 119 ± 9.5 mmHg), reduced vascular relaxation, and increased contraction compared to WT mice (see Supplementary material online, *Figure S14A*–*C*). IFNλ3, IFNα, and SP1 reduced endothelium-dependent relaxation in mesenteric vessels from WT mice as observed by a decrease in maximal responses to acetylcholine (Ach) (*Figure 7A* and *B*). No effects were observed on contraction induced by U446619 or endothelial-independent relaxation induced by sodium nitroprusside (see Supplementary material online, *Figure S14D*–*G*). IFNα increased sensitivity to contraction in vessels from LinA3 mice (see Supplementary material online, *Figure S14H*). No effects were observed on endothelium-dependent or endothelium-independent relaxation in vessels from LinA3 mice stimulated with IFNs or SP1 (*Figure 7C and D* and Supplementary material online, *Figure S14I and K*).

To investigate the potential role of ISG15 in IFN-induced vascular effects, we assessed vascular function in mesenteric vessels from ISG15−/− and WT (ISG15+/+) mice. These mice exhibited similar Ach-induced relaxation and U46619-dependent contraction (see Supplementary material online, *Figure S15A* and *B*). Reduced endothelium-dependent relaxation was observed in ISG+/+ mice treated with IFNλ3 and IFNα, effects that were abrogated in vessels from ISG15−/− mice (*Figure 7E and G*). Vessels from ISG15−/− mice treated with IFNα and IFNλ3 exhibited reduced sensitivity to contraction induced by U46619 (see Supplementary material online, *Figure S15D*). However, no changes in vascular function were observed in ISG15−/− or ISG15+/+ mice treated with SP1 (see Supplementary material online, *Figure S15E and F*). These differences might be due to distinct mice strains. LinA3 and WT are derived under FVB background, whereas ISG15+/+ and ISG15−/− are derived under C57Bl/6 background. To further investigate the role of ISG15 in the context of COVID-19, we used the full recombinant spike protein of SARS-CoV-2, which reduced endothelial-dependent relaxation in vessels from ISG15+/+ mice (*Figure 7F*). These effects were abrogated in ISG15−/− mice (*Figure 7H*). Our findings indicate that endothelial dysfunction induced by IFNα, IFNλ3 and spike protein is dependent on the activation of ISG15.

## 4. Discussion

In the present study, we demonstrate that SP1 of SARS-CoV-2 induces an inflammatory response in microvascular ECs by increasing the expression of ISGs, especially ISG15, effects that were abrogated by ADAM17 and STAT1 inhibitors. In particular, we show that SP1-stimulated ECs mount an immune/inflammatory response by increasing expression of IFNλ3 and IFNα, which are important in viral infection. This is associated with decreased EC NO production and endothelial dysfunction by mechanisms dependent on ISG15. In the context of hypertension, IFN-ISG15 increased vascular reactivity. Together, our study demonstrates that ECs participate in the immune/inflammatory response to SP1 and expression of IFN-stimulated cardiovascular sequelae of the infection.

Type I and Type III IFNs are crucial mediators of the innate immune response against viral infection, and have been observed in SARS-CoV-2-infected patients.^1,28^ Additionally, increased expression of ISGs has been demonstrated in ECs from skin biopsies from COVID-19 patients.^29^ Here, we focus on IFNα and IFNλ3 as examples of type I and type III IFN, respectively. Microvascular ECs express ISGs (ISG15, MX1, and IFIT1) in response to SP1, IFNα, and IFNλ3 by mechanisms dependent on ADAM17 and STAT1 activation. IFNλ3 failed to induce effects in pulmonary, lymphatic, and aortic EC, suggesting that the response of EC to IFNs is not a generalized EC phenomenon but IFN-and cell-specific. Such differences in cell activation in response to IFNλ3 might result from distinct signalling pathways, as IFNλ3 induced phosphorylation of STAT2, ERK1/2, and P38MAPK only in microvascular ECs and not in lymphatic ECs. Although both cell populations express similar levels of IFNLR1.

In addition to vaccines, other therapeutic approaches have been considered in the management of COVID19, including the use of IFNs.^3–5^ This may be especially important in immunocompromised patients.^30^ Of importance, IFNs are also considered immunotherapeutic opportunities in cancer treatment.^31^ However, it is unclear whether these IFNs may have potential deleterious effects. We found that IFNα and IFNλ3 induce phosphorylation of pro-inflammatory signalling molecules STAT1, STAT2, and ERK1/2 and stimulate ISG expression. These effects were amplified by SP1. While IFNα tended to have greater responses, both IFNs induced IL-6 and TNFα production and PAI-1 expression, which are pro-inflammatory mediators with direct impact on endothelial function, vascular inflammation, and thrombus formation in COVID-19 patients.^32^ Additionally, these mediators are increased in EC treated with plasma from critically ill and convalescent COVID-19 patients.^33^ Here we advance this knowledge and indicate that IFNα and IFNλ3 may also be involved in EC inflammation and vascular dysfunction during SARS-CoV-2 infection.

The main mechanism of SARS-CoV-2 entry involves direct interaction of the SP1 protein with ACE2 in host cells and the crucial participation of cell proteases, including TMPRSS2 and ADAM17.^8,9^ ACE2 enzyme is a component of the counter-regulatory axis of the renin-angiotensin system since it generates Ang-1-7, a vasoprotective and anti-inflammatory peptide. Binding of SARS-CoV-2 to ACE2 leads to endosome formation, reducing ACE2 expression and possibly decreasing Ang-(1-7) formation,^34^ driving the system to a pro-inflammatory phenotype,^14,35^ which together with increased IFNs, IL-6, TNFα, adhesion molecules, and PAI-1 induces endothelial inflammation and vascular injury. We previously showed that this inflammatory response is independent of viral replication.^14^ Here we show some additional damaging vascular effects induced by SP1 such as decreased cell migration. Mechanisms underlying these responses are still elusive and might be associated with cell senescence and DNA damage.^36^

Various mechanisms whereby SARS-CoV-2 and its S1 subunit induce endothelial inflammation have been described, including activation of mineralocorticoid receptor, proteins of complement system and endothelial cell damaging by neutrophil extracellular traps (NETs).^14,37–40^ Virus internalization leads to activation of several intracellular inflammatory pathways, including Toll-like receptor (TLR) -3, -7, and -8.^41,42^ Membrane receptors are also activated, including TLR-2 and -4 that are involved in the inflammatory response by mechanisms that are independent of virus internalization.^43,44^ Here we advance the notion and show that SP1 stimulates expression of the IFN response genes ISG15, MX1, and IFIT1 in microvascular EC, effects that were amplified in the presence of IFNs. These findings suggest synergic effects of SP1 and IFNs that may aggravate the inflammatory response and vascular damage. Of importance, such effects were not observed in lymphatic EC. Upregulation of TMPRSS2 and ADAM17 is associated with increased susceptibility to SARS-CoV-2 infection^9^ and we found that both were increased by IFNs in EC. TMPRSS2 inhibition reduces SARS-CoV-2 infection^45^ probably by preventing SP1 priming and ACE2 interaction. Here, we show that inhibition of ADAM17 reduced expression of ISGs induced by SP1. These effects might involve STAT1 activation, since SP1 failed to increase ISGs in the presence of STAT1 inhibition and in STAT1-deficient cells. Increased ADAM17 activity is associated with chronic pulmonary inflammation, hypertension, renal, and cardiovascular disease.^10,46^ In the context of SARS-CoV-2 infection, ADAM17 activation induces shedding of ACE2 favouring cytokine production, including TNFα, IFNγ, and IL-1β in epithelial cells.^47^ Together, our results demonstrate that microvascular ECs exhibit high expression of ADAM17, which regulates expression of SP1-induced ISGs ISG15, MX1 and IFIT1. Mechanisms involved in this activation are still unclear but might involve of STAT1, since SP1 induces production of IFNs, which in turn activate expression of ISGs. In addition, ADAM17 contributes to STAT1 activation.^48^ Our data, using pharmacologic inhibitors and STAT1-deficient A495 cells, confirmed the crucial role of STAT1 in SP1 regulation of ISG15, MX1, and IFIT1. Our findings support others in human macrophages, where ADAM17 inhibition reduces the effects of IFNα.^49^ Hence exacerbated ADAM17 activity emerges as a possible underlying mechanism for the acute inflammatory immune response in COVID-19. Moreover, experiments using organoid models and ACE2 transgenic mice demonstrated that cytokine production induced by SARS-CoV-2 infection contributes to cardiac dysfunction in long COVID patients.^50^

To advance the concept that IFNs may influence vascular function in COVID-19,^51^ we investigated vascular effects of IFNα and IFNλ3 in intact vessels. Endothelium-dependent vasorelaxation was reduced by IFNα, IFNλ3 or SP1, while hypercontractile responses to the vasoconstrictor U446619 were observed only in arteries from hypertensive mice that were exposed to IFNα. Our previous studies identified ISG15 as an important player in vascular dysfunction in hypertension since ISG15 is increased in aortas from hypertensive animals and in peripheral blood mononuclear cells from hypertensive humans,^52^ whereas ISG15-deficient animals exhibited reduced blood pressure and improved vascular function.^52^ Here we advance these concepts and show that ISG15 may also be important in vascular injury in the context of hypertension and SARS-CoV-2 infection, since IFNα, IFNλ3, or recombinant spike protein-induced endothelial dysfunction was abrogated in ISG15−/− mice. While our *in vivo* studies do not allow us to confirm if the vascular dysfunction is due to an endothelium-specific mechanism, additional studies in mice with endothelial cell-specific ISG15 knockout will provide greater insights. Furthermore, whether these effects contribute to post-COVID-19 sequelae, such as chronic vascular inflammation and blood pressure changes, is still unknown and warrants further investigations.

Our findings need to be considered within the context of some limitations of the study, which was conducted using *in vitro* and *ex vivo* approaches. In vitro studies were performed in primary cultured ECs. Although these cells are appropriate for studying molecular and cellular mechanisms associated with vascular homeostasis, we cannot confirm the physiological significance of such cell models in human hypertension. Additionally, while these ECs were isolated from humans and cultured with appropriate medium as recommended by the manufacturer, we cannot guarantee that they were uniquely isolated from arteries and/or arterioles, without potential contamination by ECs from other vessels, such as capillaries. Nevertheless, ECs do provide a suitable and well-used model to dissect complex signalling pathways, which was the purpose in our study. We acknowledge that *in vivo* experiments performed in conditional knockout animals targeting the endothelium would help to understand the endothelial-specific role of IFNs and the impact on the intact cardiovascular system, especially in models exposed to SP1.

Another important consideration relates to our *in vitro* studies conducted with pharmacologic inhibitors. We acknowledge that there may be some non-specificity with these agents. To mitigate this, we used agents and protocols that have been well described in the literature, and in some experiments, we utilized multiple inhibitors targeting the same protein. In addition, we employed a gene-targeting strategy using siRNA gene silencing. Glycosylation motifs play an important function on SARS-CoV-2 infectivity and cell activation.^53^ In our experiments, we used a recombinant SP1 subunit derived in *Escherichia coli* expression systems. Although this approach generates proteins with low glycosylation motifs, we found increased inflammatory responses in EC. We also acknowledge the possibility that SP1 generated in procaryotic vs. eucaryotic systems might induce different inflammatory responses, and probably comparative experiments are necessary to understand possible differences. However, similar protein expression systems were used in previous experiments *in vivo*^54^ and *in vitro*,^38,55^ inducing cell activation, inflammatory response, and organ injury, justifying the strategy we employed in our studies.

In conclusion, we identify a novel pathway linking SP1, IFNs, ADAM17, and ISG15 in EC and demonstrate that SP1 and IFNs synergically induce expression of ISGs and activation of inflammatory pathways through ADAM17 and STAT1. Moreover, IFNλ3, IFNα, and SP1 induce endothelial inflammation and vascular dysfunction through ISG15-dependent processes. These IFN effects may play a role in the endotheliopathy and vascular damage associated with S1 of SARS-CoV-2 and might contribute to cardiovascular sequelae, including vascular dysfunction in hypertension, observed in patients with COVID-19. The potential deleterious vascular effects of IFNs in the vasculature warrant further consideration regarding IFN therapeutic strategies in SARS-CoV-2 infection.

## Supplementary Material

cvag111_Supplementary_Data

## Data Availability

All data are incorporated into the article and its online supplementary material. Additional material are available on request.

## References

[1] Park A, Iwasaki A. Type I and type III interferons - induction, signaling, evasion, and application to combat COVID-19. Cell Host Microbe 2020;27:870–878. 32464097 10.1016/j.chom.2020.05.008 PMC7255347

[2] Kim YM, Shin EC. Type I and III interferon responses in SARS-CoV-2 infection. Exp Mol Med 2021;53:750–760. 33953323 10.1038/s12276-021-00592-0 PMC8099704

[3] Major J, Crotta S, Llorian M, McCabe TM, Gad HH, Priestnall SL, Hartmann R, Wack A. Type I and III interferons disrupt lung epithelial repair during recovery from viral infection. Science 2020;369:712–717. 32527928 10.1126/science.abc2061 PMC7292500

[4] Shahbazi M, Amri Maleh P, Bagherzadeh M, Moulana Z, Sepidarkish M, Rezanejad M, Mirzakhani M, Ebrahimpour S, Ghorbani H, Ahmadnia Z, Javanian M, Bayani M, Mohammadnia-Afrouzi M. Linkage of lambda interferons in protection against severe COVID-19. J Interferon Cytokine Res 2021;41:149–152. 33885337 10.1089/jir.2020.0187

[5] Reis G, Moreira Silva EAS, Medeiros Silva DC, Thabane L, Campos VHS, Ferreira TS, Santos CVQ, Nogueira AMR, Almeida A, Savassi LCM, Figueiredo-Neto AD, Dias ACF, Freire Junior AM, Bitaraes C, Milagres AC, Callegari ED, Simplicio MIC, Ribeiro LB, Oliveira R, Harari O, Wilson LA, Forrest JI, Ruton H, Sprague S, McKay P, Guo CM, Limbrick-Oldfield EH, Kanters S, Guyatt GH, Rayner CR, Kandel C, Biondi MJ, Kozak R, Hansen B, Zahoor MA, Arora P, Hislop C, Choong I, Feld JJ, Mills EJ, Glenn JS; TOGETHER Investigators. Early treatment with pegylated interferon lambda for COVID-19. N Engl J Med 2023;388:518–528. 36780676 10.1056/NEJMoa2209760 PMC9933926

[6] Lee JS, Park S, Jeong HW, Ahn JY, Choi SJ, Lee H, Choi B, Nam SK, Sa M, Kwon JS, Jeong SJ, Lee HK, Park SH, Park SH, Choi JY, Kim SH, Jung I, Shin EC. Immunophenotyping of COVID-19 and influenza highlights the role of type I interferons in development of severe COVID-19. Sci Immunol 2020;5:. 10.1126/sciimmunol.abd1554 PMC7402635 32651212

[7] Bastard P, Rosen LB, Zhang Q, Michailidis E, Hoffmann HH, Zhang Y, Dorgham K, Philippot Q, Rosain J, Beziat V, Manry J, Shaw E, Haljasmagi L, Peterson P, Lorenzo L, Bizien L, Trouillet-Assant S, Dobbs K, de Jesus AA, Belot A, Kallaste A, Catherinot E, Tandjaoui-Lambiotte Y, Le Pen J, Kerner G, Bigio B, Seeleuthner Y, Yang R, Bolze A, Spaan AN, Delmonte OM, Abers MS, Aiuti A, Casari G, Lampasona V, Piemonti L, Ciceri F, Bilguvar K, Lifton RP, Vasse M, Smadja DM, Migaud M, Hadjadj J, Terrier B, Duffy D, Quintana-Murci L, van de Beek D, Roussel L, Vinh DC, Tangye SG, Haerynck F, Dalmau D, Martinez-Picado J, Brodin P, Nussenzweig MC, Boisson-Dupuis S, Rodriguez-Gallego C, Vogt G, Mogensen TH, Oler AJ, Gu J, Burbelo PD, Cohen JI, Biondi A, Bettini LR, D'Angio M, Bonfanti P, Rossignol P, Mayaux J, Rieux-Laucat F, Husebye ES, Fusco F, Ursini MV, Imberti L, Sottini A, Paghera S, Quiros-Roldan E, Rossi C, Castagnoli R, Montagna D, Licari A, Marseglia GL, Duval X, Ghosn J; HGID Lab; NIAID-USUHS Immune Response to COVID Group; COVID Clinicians; COVID-STORM Clinicians; Imagine COVID Group; French COVID Cohort Study Group; Milieu Intérieur Consortium; CoV-Contact Cohort; Amsterdam UMC Covid-19 Biobank; COVID Human Genetic Effort, Tsang JS, Goldbach-Mansky R, Kisand K, Lionakis MS, Puel A, Zhang SY, Holland SM, Gorochov G, Jouanguy E, Rice CM, Cobat A, Notarangelo LD, Abel L, Su HC and Casanova JL. Autoantibodies against type I IFNs in patients with life-threatening COVID-19. Science 2020;370:eabd4585.32972996

[8] Sungnak W, Huang N, Becavin C, Berg M, Queen R, Litvinukova M, Talavera-Lopez C, Maatz H, Reichart D, Sampaziotis F, Worlock KB, Yoshida M, Barnes JL and HCA Lung Biological Network. SARS-CoV-2 entry factors are highly expressed in nasal epithelial cells together with innate immune genes. Nat Med 2020;26:681–687. 32327758 10.1038/s41591-020-0868-6 PMC8637938

[9] Zipeto D, Palmeira JDF, Arganaraz GA, Arganaraz ER. ACE2/ADAM17/TMPRSS2 interplay may be the main risk factor for COVID-19. Front Immunol 2020;11:576745. 33117379 10.3389/fimmu.2020.576745 PMC7575774

[10] Kawai T, Elliott KJ, Scalia R, Eguchi S. Contribution of ADAM17 and related ADAMs in cardiovascular diseases. Cell Mol Life Sci 2021;78:4161–4187. 33575814 10.1007/s00018-021-03779-w PMC9301870

[11] Varga Z, Flammer AJ, Steiger P, Haberecker M, Andermatt R, Zinkernagel AS, Mehra MR, Schuepbach RA, Ruschitzka F, Moch H. Endothelial cell infection and endotheliitis in COVID-19. Lancet 2020;395:1417–1418. 32325026 10.1016/S0140-6736(20)30937-5 PMC7172722

[12] Brauninger H, Stoffers B, Fitzek ADE, Meissner K, Aleshcheva G, Schweizer M, Weimann J, Rotter B, Warnke S, Edler C, Braun F, Roedl K, Scherschel K, Escher F, Kluge S, Huber TB, Ondruschka B, Schultheiss HP, Kirchhof P, Blankenberg S, Puschel K, Westermann D, Lindner D. Cardiac SARS-CoV-2 infection is associated with pro-inflammatory transcriptomic alterations within the heart. Cardiovasc Res 2022;118:542–555. 34647998 10.1093/cvr/cvab322 PMC8803085

[13] Wang L, Sievert D, Clark AE, Lee S, Federman H, Gastfriend BD, Shusta EV, Palecek SP, Carlin AF, Gleeson JG. A human three-dimensional neural-perivascular ‘assembloid’ promotes astrocytic development and enables modeling of SARS-CoV-2 neuropathology. Nat Med 2021;27:1600–1606. 34244682 10.1038/s41591-021-01443-1 PMC8601037

[14] Montezano AC, Camargo LL, Mary S, Neves KB, Rios FJ, Stein R, Lopes RA, Beattie W, Thomson J, Herder V, Szemiel AM, McFarlane S, Palmarini M, Touyz RM. SARS-CoV-2 spike protein induces endothelial inflammation via ACE2 independently of viral replication. Sci Rep 2023;13:14086. 37640791 10.1038/s41598-023-41115-3 PMC10462711

[15] Merad M, Martin JC. Pathological inflammation in patients with COVID-19: a key role for monocytes and macrophages. Nat Rev Immunol 2020;20:355–362. 32376901 10.1038/s41577-020-0331-4 PMC7201395

[16] Alfaro E, Diaz-Garcia E, Garcia-Tovar S, Galera R, Casitas R, Torres-Vargas M, Lopez-Fernandez C, Anon JM, Garcia-Rio F, Cubillos-Zapata C. Endothelial dysfunction and persistent inflammation in severe post-COVID-19 patients: implications for gas exchange. BMC Med 2024;22:242. 38867241 10.1186/s12916-024-03461-5 PMC11170912

[17] Faria D, Moll-Bernardes RJ, Testa L, Moniz CMV, Rodrigues EC, Rodrigues AG, Araujo A, Alves M, Ono BE, Izaias JE, Salemi VMC, Jordao CP, Amaro-Vicente G, Rondon M, Ludwig KR, Craighead DH, Rossman MJ, Consolim-Colombo FM, De Angelis K, Irigoyen MCC, Seals DR, Negrao CE, Sales ARK. Sympathetic neural overdrive, aortic stiffening, endothelial dysfunction, and impaired exercise capacity in severe COVID-19 survivors: a mid-term study of cardiovascular sequelae. Hypertension 2023;80:470–481. 36416143 10.1161/HYPERTENSIONAHA.122.19958 PMC9847692

[18] Shibata S, Kobayashi K, Tanaka M, Asayama K, Yamamoto E, Nakagami H, Hoshide S, Kishi T, Matsumoto C, Mogi M, Morimoto S, Yamamoto K, Mukoyama M, Kario K, Node K, Rakugi H. COVID-19 pandemic and hypertension: an updated report from the Japanese society of hypertension project team on COVID-19. Hypertens Res 2023;46:589–600. 36550205 10.1038/s41440-022-01134-5 PMC9780104

[19] Zhang V, Fisher M, Hou W, Zhang L, Duong TQ. Incidence of new-onset hypertension post-COVID-19: comparison with influenza. Hypertension 2023;80:2135–2148. 37602375 10.1161/HYPERTENSIONAHA.123.21174

[20] Gonzalez-Amor M, Garcia-Redondo AB, Jorge I, Zalba G, Becares M, Ruiz-Rodriguez MJ, Rodriguez C, Bermeo H, Rodrigues-Diez R, Rios FJ, Montezano AC, Martinez-Gonzalez J, Vazquez J, Redondo JM, Touyz RM, Guerra S, Salaices M, Briones AM. Interferon stimulated gene 15 pathway is a novel mediator of endothelial dysfunction and aneurysms development in angiotensin II infused mice through increased oxidative stress. Cardiovasc Res 2022;118:3250–3268. 34672341 10.1093/cvr/cvab321 PMC9799052

[21] Ran FA, Hsu PD, Wright J, Agarwala V, Scott DA, Zhang F. Genome engineering using the CRISPR-cas9 system. Nat Protoc 2013;8:2281–2308. 24157548 10.1038/nprot.2013.143 PMC3969860

[22] Avolio E, Carrabba M, Milligan R, Kavanagh Williamson M, Beltrami AP, Gupta K, Elvers KT, Gamez M, Foster RR, Gillespie K, Hamilton F, Arnold D, Berger I, Davidson AD, Hill D, Caputo M, Madeddu P. The SARS-CoV-2 spike protein disrupts human cardiac pericytes function through CD147 receptor-mediated signalling: a potential non-infective mechanism of COVID-19 microvascular disease. Clin Sci (Lond) 2021;135:2667–2689. 34807265 10.1042/CS20210735 PMC8674568

[23] Gultom M, Lin L, Brandt CB, Milusev A, Despont A, Shaw J, Doring Y, Luo Y, Rieben R. Sustained vascular inflammatory effects of SARS-CoV-2 spike protein on human endothelial cells. Inflammation 2024;48:2531–2547. 39739157 10.1007/s10753-024-02208-x PMC12336097

[24] Li CJ, Chang SC. SARS-CoV-2 spike S2-specific neutralizing antibodies. Emerg Microbes Infect 2023;12:2220582. 37254830 10.1080/22221751.2023.2220582 PMC10274517

[25] Prescott G, Silversides DW, Chiu SM, Reudelhuber TL. Contribution of circulating renin to local synthesis of angiotensin peptides in the heart. Physiol Genomics 2000;4:67–73. 11074015 10.1152/physiolgenomics.2000.4.1.67

[26] Touyz RM, Mercure C, He Y, Javeshghani D, Yao G, Callera GE, Yogi A, Lochard N, Reudelhuber TL. Angiotensin II-dependent chronic hypertension and cardiac hypertrophy are unaffected by gp91phox-containing NADPH oxidase. Hypertension 2005;45:530–537. 15753233 10.1161/01.HYP.0000158845.49943.5e

[27] Burger D, Reudelhuber TL, Mahajan A, Chibale K, Sturrock ED, Touyz RM. Effects of a domain-selective ACE inhibitor in a mouse model of chronic angiotensin II-dependent hypertension. Clin Sci (Lond) 2014;127:57–63. 24506807 10.1042/CS20130808

[28] Sposito B, Broggi A, Pandolfi L, Crotta S, Clementi N, Ferrarese R, Sisti S, Criscuolo E, Spreafico R, Long JM, Ambrosi A, Liu E, Frangipane V, Saracino L, Bozzini S, Marongiu L, Facchini FA, Bottazzi A, Fossali T, Colombo R, Clementi M, Tagliabue E, Chou J, Pontiroli AE, Meloni F, Wack A, Mancini N, Zanoni I. The interferon landscape along the respiratory tract impacts the severity of COVID-19. Cell 2021;184:4953–4968.e16. 34492226 10.1016/j.cell.2021.08.016 PMC8373821

[29] Di Domizio J, Gulen MF, Saidoune F, Thacker VV, Yatim A, Sharma K, Nass T, Guenova E, Schaller M, Conrad C, Goepfert C, De Leval L, von Garnier C, Berezowska S, Dubois A, Gilliet M, Ablasser A. The cGAS-STING pathway drives type I IFN immunopathology in COVID-19. Nature 2022;603:145–151. 35045565 10.1038/s41586-022-04421-w PMC8891013

[30] Yong MK, Thursky K, Crane M, Spelman T, Mahar RK, Simpson JA, Scott AM, Harrison SJ, Szer J, Pellegrini M, Lingaratnam S, Pang KC, Tennakoon S, Sim BZ, Blyth E, Gan HK, Quach H, McIntosh MP, Page H, Woolstencroft R, Slavin M. Interferon-alpha nasal spray prophylaxis reduces COVID-19 in cancer patients: a randomized, double-blinded, placebo-controlled trial. Clin Infect Dis 2025;82:e208–e216. 10.1093/cid/ciaf409 PMC13017443 40874769

[31] Borden EC . Interferons alpha and beta in cancer: therapeutic opportunities from new insights. Nat Rev Drug Discov 2019;18:219–234. 30679806 10.1038/s41573-018-0011-2

[32] Lucas C, Wong P, Klein J, Castro TBR, Silva J, Sundaram M, Ellingson MK, Mao T, Oh JE, Israelow B, Takahashi T, Tokuyama M, Lu P, Venkataraman A, Park A, Mohanty S, Wang H, Wyllie AL, Vogels CBF, Earnest R, Lapidus S, Ott IM, Moore AJ, Muenker MC, Fournier JB, Campbell M, Odio CD, Casanovas-Massana A, Yale IT, Herbst R, Shaw AC, Medzhitov R, Schulz WL, Grubaugh ND, Dela Cruz C, Farhadian S, Ko AI, Omer SB, Iwasaki A. Longitudinal analyses reveal immunological misfiring in severe COVID-19. Nature 2020;584:463–469. 32717743 10.1038/s41586-020-2588-y PMC7477538

[33] Rauch A, Dupont A, Goutay J, Caplan M, Staessens S, Moussa M, Jeanpierre E, Corseaux D, Lefevre G, Lassalle F, Faure K, Lambert M, Duhamel A, Labreuche J, Garrigue D, De Meyer SF, Staels B, Van Belle E, Vincent F, Kipnis E, Lenting PJ, Poissy J, Susen S; Lille COVID Research Network (LICORNE); Members of the LICORNE Scientific Committee. Endotheliopathy is induced by plasma from critically ill patients and associated with organ failure in severe COVID-19. Circulation 2020;142:1881–1884. 32970476 10.1161/CIRCULATIONAHA.120.050907 PMC7643783

[34] Lei Y, Zhang J, Schiavon CR, He M, Chen L, Shen H, Zhang Y, Yin Q, Cho Y, Andrade L, Shadel GS, Hepokoski M, Lei T, Wang H, Zhang J, Yuan JX, Malhotra A, Manor U, Wang S, Yuan ZY, Shyy JY. SARS-CoV-2 spike protein impairs endothelial function via downregulation of ACE 2. Circ Res 2021;128:1323–1326. 33784827 10.1161/CIRCRESAHA.121.318902 PMC8091897

[35] Dolci M, Signorini L, D'Alessandro S, Perego F, Parapini S, Sommariva M, Taramelli D, Ferrante P, Basilico N, Delbue S. In vitro SARS-CoV-2 infection of microvascular endothelial cells: effect on pro-inflammatory cytokine and chemokine release. Int J Mol Sci 2022;23:4063. 35409421 10.3390/ijms23074063 PMC8999888

[36] Villacampa A, Shamoon L, Valencia I, Morales C, Figueiras S, de la Cuesta F, Sanchez-Nino D, Diaz-Araya G, Sanchez-Perez I, Lorenzo O, Sanchez-Ferrer CF, Peiro C. SARS-CoV-2 S protein reduces cytoprotective defenses and promotes human endothelial cell senescence. Aging Dis 2024;16:1626–1638. 39012668 10.14336/AD.2024.0405 PMC12096926

[37] Kumar N, Zuo Y, Yalavarthi S, Hunker KL, Knight JS, Kanthi Y, Obi AT, Ganesh SK. SARS-CoV-2 spike protein S1-mediated endothelial injury and pro-inflammatory state is amplified by dihydrotestosterone and prevented by mineralocorticoid antagonism. Viruses 2021;13:2209. 34835015 10.3390/v13112209 PMC8617813

[38] Rotoli BM, Barilli A, Visigalli R, Ferrari F, Dall'Asta V. Endothelial cell activation by SARS-CoV-2 spike S1 protein: a crosstalk between endothelium and innate immune cells. Biomedicines 2021;9:1220. 34572407 10.3390/biomedicines9091220 PMC8470710

[39] Perico L, Morigi M, Pezzotta A, Locatelli M, Imberti B, Corna D, Cerullo D, Benigni A, Remuzzi G. SARS-CoV-2 spike protein induces lung endothelial cell dysfunction and thrombo-inflammation depending on the C3a/C3a receptor signalling. Sci Rep 2023;13:11392. 37452090 10.1038/s41598-023-38382-5 PMC10349115

[40] Skendros P, Mitsios A, Chrysanthopoulou A, Mastellos DC, Metallidis S, Rafailidis P, Ntinopoulou M, Sertaridou E, Tsironidou V, Tsigalou C, Tektonidou M, Konstantinidis T, Papagoras C, Mitroulis I, Germanidis G, Lambris JD, Ritis K. Complement and tissue factor-enriched neutrophil extracellular traps are key drivers in COVID-19 immunothrombosis. J Clin Invest 2020;130:6151–6157. 32759504 10.1172/JCI141374 PMC7598040

[41] Mukherjee R, Bhattacharya A, Bojkova D, Mehdipour AR, Shin D, Khan KS, Hei-Yin Cheung H, Wong KB, Ng WL, Cinatl J, Geurink PP, van Noort GJ vdH, Rajalingam K, Ciesek S, Hummer G, Dikic I. Famotidine inhibits toll-like receptor 3-mediated inflammatory signaling in SARS-CoV-2 infection. J Biol Chem 2021;297:100925. 34214498 10.1016/j.jbc.2021.100925 PMC8241579

[42] Bortolotti D, Gentili V, Rizzo S, Schiuma G, Beltrami S, Strazzabosco G, Fernandez M, Caccuri F, Caruso A, Rizzo R. TLR3 and TLR7 RNA sensor activation during SARS-CoV-2 infection. Microorganisms 2021;9:1820. 34576716 10.3390/microorganisms9091820 PMC8465566

[43] Zheng M, Karki R, Williams EP, Yang D, Fitzpatrick E, Vogel P, Jonsson CB, Kanneganti TD. TLR2 senses the SARS-CoV-2 envelope protein to produce inflammatory cytokines. Nat Immunol 2021;22:829–838. 33963333 10.1038/s41590-021-00937-x PMC8882317

[44] Zhao Y, Kuang M, Li J, Zhu L, Jia Z, Guo X, Hu Y, Kong J, Yin H, Wang X, You F. Publisher correction: SARS-CoV-2 spike protein interacts with and activates TLR4. Cell Res 2021;31:825. 33907310 10.1038/s41422-021-00501-0 PMC8077189

[45] Hoffmann M, Kleine-Weber H, Schroeder S, Kruger N, Herrler T, Erichsen S, Schiergens TS, Herrler G, Wu NH, Nitsche A, Muller MA, Drosten C, Pohlmann S. SARS-CoV-2 cell entry Depends on ACE2 and TMPRSS2 and is blocked by a clinically proven protease inhibitor. Cell 2020;181:271–280.e8. 32142651 10.1016/j.cell.2020.02.052 PMC7102627

[46] Saad MI, McLeod L, Hodges C, Vlahos R, Rose-John S, Ruwanpura S, Jenkins BJ. ADAM17 deficiency protects against pulmonary emphysema. Am J Respir Cell Mol Biol 2021;64:183–195. 33181031 10.1165/rcmb.2020-0214OC

[47] Mattos Pereira V, Thakar A, Nair S. Targeting iRhom2/ADAM17 attenuates COVID-19-induced cytokine release from cultured lung epithelial cells. Biochem Biophys Rep 2024;39:101811. 39253056 10.1016/j.bbrep.2024.101811 PMC11382212

[48] Li M, Xue W, Li X, Song Y, Liu X, Qin L. Axl is related to inflammation in hemodialysis patients. Mol Immunol 2021;133:146–153. 33667984 10.1016/j.molimm.2021.02.024

[49] Anthony SM, Howard ME, Hailemichael Y, Overwijk WW, Schluns KS. Soluble interleukin-15 complexes are generated in vivo by type I interferon dependent and independent pathways. PLoS One 2015;10:e0120274. 25756182 10.1371/journal.pone.0120274 PMC4354909

[50] Thomas D, Noishiki C, Gaddam S, Wu D, Manhas A, Liu Y, Tripathi D, Kathale N, Adkar SS, Garhyan J, Liu C, Xu B, Ross EG, Dalman RL, Wang KC, Oro AE, Sallam K, Lee JT, Wu JC, Sayed N. CCL2-mediated endothelial injury drives cardiac dysfunction in long COVID. Nat Cardiovasc Res 2024;3:1249–1265. 39402206 10.1038/s44161-024-00543-8 PMC12243935

[51] Evans PC, Rainger GE, Mason JC, Guzik TJ, Osto E, Stamataki Z, Neil D, Hoefer IE, Fragiadaki M, Waltenberger J, Weber C, Bochaton-Piallat ML, Back M. Endothelial dysfunction in COVID-19: a position paper of the ESC working group for atherosclerosis and vascular biology, and the ESC council of basic cardiovascular science. Cardiovasc Res 2020;116:2177–2184. 32750108 10.1093/cvr/cvaa230 PMC7454368

[52] Ostrowska A, Wojciechowska W, Rajzer M, Weber T, Bursztyn M, Persu A, Stergiou G, Kielbasa G, Chrostowska M, Doumas M, Parati G, Bilo G, Grassi G, Mancia G, Januszewicz A, Kreutz R; ESH ABPM COVID-19 Study Investigators. The impact of the COVID-19 pandemic on hypertension phenotypes (ESH ABPM COVID-19 study). Eur J Intern Med 2025;131:58–64. 39261181 10.1016/j.ejim.2024.08.027

[53] Gong Y, Qin S, Dai L, Tian Z. The glycosylation in SARS-CoV-2 and its receptor ACE2. Signal Transduct Target Ther 2021;6:396. 34782609 10.1038/s41392-021-00809-8 PMC8591162

[54] Biancatelli RML C, Solopov PA, Sharlow ER, Lazo JS, Marik PE, Catravas JD. The SARS-CoV-2 spike protein subunit S1 induces COVID-19-like acute lung injury in Kappa18-hACE2 transgenic mice and barrier dysfunction in human endothelial cells. Am J Physiol Lung Cell Mol Physiol 2021;321:L477–L484. 34156871 10.1152/ajplung.00223.2021 PMC8384477

[55] Perico L, Morigi M, Galbusera M, Pezzotta A, Gastoldi S, Imberti B, Perna A, Ruggenenti P, Donadelli R, Benigni A, Remuzzi G. SARS-CoV-2 spike protein 1 activates microvascular endothelial cells and complement system leading to platelet aggregation. Front Immunol 2022;13:827146. 35320941 10.3389/fimmu.2022.827146 PMC8936079

