# Supplementary material for: Spike 1 protein of SARS-CoV-2 induces endothelial inflammation and vascular dysfunction through interferon ISG15-dependent mechanisms
Source: Cardiovasc Res. 2026 May 18;122(10):1344–58. doi: 10.1093/cvr/cvag111 (PMC13355842; doi:10.1093/cvr/cvag111)

SUPPLEMENTAL TEXT

Spike 1 protein of SARS-CoV-2 induces endothelial inflammation and vascular dysfunction through interferon ISG15-dependent mechanisms

Francisco J. Rios[1 2], Augusto C Montezano[1], Livia L Camargo[1], Rheure A Lopes[3], Ana B. García-Redondo[4 5 6], Elihu Aranday-Cortes[7], Ana M Briones[6 8], John McLauchlan[7],

Rhian M Touyz[1,9].

[1] Research Institute of the McGill University Health Centre, Montreal, Canada; ; [2] School of Medicine, Pharmacy and Biomedical Sciences, University of Portsmouth, Portsmouth, UK; [3] Institute of Cardiovascular and Medical Sciences, University of Glasgow, Glasgow, UK; [4]Departmento de Fisiología, Facultad de Medicina, Universidad Autónoma de Madrid, Madrid, Spain; [5]Instituto de Investigación Hospital La Paz (IdiPAZ), Madrid, Spain; [6] Centro de Investigación Biomédica en Red de Enfermedades Cardiovasculares, Spain; [7] MRC-Centre for Virus Research, University of Glasgow, Glasgow, United Kingdom; [8] Departamento de Farmacología, Universidad Autónoma de Madrid, Instituto de Investigación Hospital La Paz, Madrid, Spain; [9] Departments of Medicine and Department of Family Medicine, McGill University, Montreal, Canada

Key words: IFNα, IFNλ, EC, COVID-19, SARS-CoV-2

Correspondence:

*Francisco J. Rios, PhD
Pharmacy and Biomedical Sciences,
University of Portsmouth
St Michael's building, White Swan Rd
Portsmouth, UK, PO1 2DT
Email: francisco.rios@port.ac.uk

*Rhian M Touyz MBBCh, PhD
Research Institute of the McGill University Health Centre (RI-MUHC)
1001, boul Décarie, ES1.5066.6
Montréal, Québec, Canada, H4A 3J1

tél./tel. (514) 934-1934 #71608

email: rhian.touyz@mcgill.ca

Supplemental table 1. Primer sequences for human samples

	Forward 5'-3'	Reverse 5'-3'
**hGAPDH housekeeping**	GAGTCAACGGATTTGGTCGT<br>Tm 58.21	TTGATTTTGGAGGGATCTCG<br>Tm 55.13
	Specific for:<br>NM_001357943.2 – variant 7, amplicon = 184<br>NM_001357943.2 – variant 3, amplicon = 238<br>NM_001289746.2 – variant 4, amplicon = 238<br>NM_002046.7 – variant 1, amplicon = 238<br>No unspecific results were found at Primer-Blast database with similar amplicon sizes.	
**hMX1**	AGATCCAGGACCAGCTGAGCCTGT<br>Tm 67.15	GTGGAACTCGTGTCGGAGTCTGG<br>TA<br>Tm 65.69
	Specific for:<br>NM_001144925.2 – variant 1, amplicon = 328<br>NM_002462.5 – variant 2, amplicon = 328<br>NM_001178046.3 – variant 3, amplicon = 328<br>NM_001282920.1 – variant 4, amplicon = 328<br>Primers are also specific for all others predicted variants with a product size of 328.<br>No unspecific results were found at Primer-Blast database with similar amplicon sizes.	
**hISG15**	CGCAGATCACCCAGAAGATCG<br>Tm 60.87	TTCGTCGCATTTGTCCACCA<br>Tm 60.53
	Specific for:<br>NM_005101.4 – amplicon = 152<br>No unspecific results were found at Primer-Blast database with similar amplicon sizes.	
**hIFIT1**	TGACTCTTTTGCCTCTTTCTTCTAA<br>Tm 58.41	TTCTTGGGGTGCTCTGTGG<br>Tm 59.54
	Specific for:<br>NM_001548.5 – variant 1, amplicon = 204<br>NM_001270927.2 – variant 2, amplicon = 204<br>NM_001270928.2 – variant 3, amplicon = 204<br>NM_001270929.2 – variant 4, amplicon = 204<br>NM_001270930.2 – variant 5, amplicon = 204<br>No unspecific results were found at Primer-Blast database with similar amplicon sizes.	
**hIFNλ2/3**	GCCACATAGCCCAGTTCAAG<br>Tm 58.90	TGGGAGAGGATATGGTGCAG<br>Tm 58.57
	Specific for:<br>NM_001548.5 – IFNL3 variant 1, amplicon = 286<br>NM_172139.4 – IFNL3 variant 2, amplicon = 286<br>NM_172138.2– IFNL2, amplicon = 286<br>No unspecific results were found at Primer-Blast database with similar amplicon sizes.	
**hIFNλ4**	TTGGCTTCCCTGACGTCTCT<br>Tm 55.00	CTCTTCCTCGTAGCGGTCCC<br>Tm 65.00

	Specific for:<br>NM_001276254.2 – amplicon = 199<br>No unspecific results were found at Primer-Blast database with similar amplicon sizes.	
**hIFNβ**	GCCGCATTGACCATCTAT<br>Tm 54.84	GTCTCATTCCAGCCAGTG<br>Tm 55.03
	Specific for:<br>NM_002176.4 – IFNB1, amplicon = 83<br>No unspecific results were found at Primer-Blast database with similar amplicon sizes.	
**hIFNα**	GACTCCATCTTGGCTGTGA<br>Tm 52.63	TGATTTCTGCTCTGACAACCT<br>Tm 42.86
	Specific for:<br>NM_006900.4 - IFNA13, amplicon = 103<br>NM_024013.3 - IFNA1, amplicon = 103<br>NM_021068.4 - IFNA4, amplicon = 103<br>NM_002172.3 - IFNA14, amplicon = 103<br>NM_002170.4 - IFNA8, amplicon = 103<br>NM_002173.3 - IFNA16, amplicon = 103<br>NM_002171.2 - IFNA10, amplicon = 103, one base misplaced at the forward primer<br>NM_021268.2 - IFNA17, amplicon = 103, one base misplaced at the forward primer<br>NM_002175.2 - IFNA21, amplicon = 103, one base misplaced at the forward primer<br>NM_021002.2 - IFNA6, amplicon = 103, one base misplaced at the forward primer<br>No unspecific results were found at Primer-Blast database with similar amplicon sizes.	
**hTNFα**	AGCCCATGTTGTAGCAAACC<br>Tm 58.74	TGAGGTACAGGCCCTCTGAT<br>Tm 59.66
	Specific for:<br>NM_000594.4 TNF, amplicon = 134<br>No unspecific results were found at Primer-Blast database with similar amplicon sizes.	
**hMCP-1 (CCL2)**	CCCCAGTCACCTGCTGTTAT<br>Tm 59.38	AGATCTCCTTGGCCACAATG<br>Tm 57.57
	Specific for:<br>NM_002982.4 – MCP-1 (CCL2), amplicon = 135<br>No unspecific results were found at Primer-Blast database with similar amplicon sizes.	
**hIL-6**	AGGAGACTTGCCTGGTGAAA<br>Tm 58.86	CAGGGGTGGTTATTGCATCT<br>Tm 57.56
	Specific for:<br>NM_001371096.1 - IL6, transcript variant 3, amplicon = 180<br>NM_001318095.2 - IL6, transcript variant 2, amplicon = 180<br>NM_000600.5 - IL6, transcript variant 1, amplicon = 180<br>No unspecific results were found at Primer-Blast database with similar amplicon sizes.	
**hIL-1β**	TCCAGGGACAGGATATGGAG<br>Tm 57.59	TCTTTCAACACGCAGGACAG<br>Tm 58.42
	Specific for:<br>NM_000576.3 - IL1B, amplicon = 133<br>No unspecific results were found at Primer-Blast database with similar amplicon sizes.	

Supplemental Figures Legends

Supplemental Figure S1. Expression of ACE2, ADAM17 and TMPRSS2 in human endothelial cells. Primary cultures of human aortic endothelial cells (AEC), pulmonary EC (PEC), microvascular EC (MEC) and lymphatic EC (LEC) were investigated for protein expression of (A) ACE2, (B) ADAM17 and (C) TMPRSS2 (n=12). Target proteins were normalized by β-actin. One-way ANOVA followed by Dunnett's multiple comparisons test were used for statistical analysis. Statistically significant p-values are shown. NS – Not Significant.

Supplemental Figure S2. Expression of ISGs and IFNs in human microvascular endothelial cells treated with spike protein 1. Microvascular endothelial cells (MEC) were treated with different concentrations of Spike Protein S1 (S1). RNA expression for (A) ISG15, (B) MX1, (C) IFIT1, (D) IFNα, (E) IFNβ, (F) IFNλ2-3 and (G) IFNλ4 was investigated after 5 h stimulation and normalized by GAPDH (n=5-8). One-way ANOVA followed by Dunnett's multiple comparisons test were used for statistical analysis. Statistically significant p-values are shown. NS – Not Significant.

Supplemental Figure S3. Expression of ISGs in human endothelial cells treated with spike protein S1 and S2. Primary cultures of (A-C) lymphatic EC (LEC) (n=6), (D-F) aortic EC (AEC) (n=6) and (G-I) pulmonary EC (PEC) (n=6) were stimulated with 1 μg/mL of spike protein 1 (S1) or spike protein 2 (S2). Gene expression for ISG15, IFIT1 and MX1 was investigated after 5 h or 24 h and normalized by GAPDH. One-way ANOVA followed by Dunnett's multiple comparisons test were used for statistical analysis. Statistically significant p-values are shown. NS – Not Significant.

Supplemental Figure S4. Expression of ISGs in human endothelial cells treated with poly(I:C). (A) Microvascular endothelial cells (MEC), lymphatic EC (LEC), aortic EC (AEC) and pulmonary (EC) were stimulated with poly(I:C) (100 ng/mL). Gene expression for ISG15, IFIT1 and MX1 was investigated after 5 h stimulation and normalized by GAPDH (n=7-8).

One-way ANOVA followed by Dunnett's multiple comparisons test were used for statistical analysis. Statistically significant p-values are shown. NS – Not Significant.

Supplemental Figure S5. Expression of ISGs in endothelial cells stimulated with Spike protein S1 of the SARS-CoV-2 (SP1) and IFNs. (A) Lymphatic endothelial cells (LEC) were treated with IFNλ3 (100 ng/mL) or IFNα (100 IU/mL) for 4 h and then treated with spike 1 protein (S1) (1 μg/mL) for additional 5 h. (B) Aortic EC (AEC) and (C) Pulmonary EC (PEC) were treated with IFNλ3 (100 ng/mL) or IFNα (100 IU/mL). Gene expression for ISG15, IFIT1 and MX1 was investigated after 5 h stimulation and normalized by GAPDH (n=7-8). One-way ANOVA followed by Dunnett's multiple comparisons test were used for statistical analysis. Individual effects were determined by a two-tailed unpaired Student's t-test. Statistically significant p-values are shown. NS – Not Significant.

Supplemental Figure S6. Expression of IFNLR1 human endothelial cells. Primary cultures of microvascular endothelial cells (MEC) and lymphatic EC (LEC) were stimulated with 1 μg/mL of spike protein 1 (S1). IFNLR1 (IFNλ3 receptor) was assessed by flow cytometry after 24 h. One-way ANOVA followed by Dunnett's multiple comparisons test were used for statistical analysis. Statistically significant p-values are shown.

Supplemental Figure S7. Expression of ACE2, TMPRSS2 and ADAM17 in human endothelial cells treated with IFNλ3 and IFNα. Primary cultures of human (A) lymphatic endothelial cells (LEC), (B) aortic EC (AEC) and (C) pulmonary EC (PEC) were treated with IFNλ3 (100 ng/mL), IFNα (100 IU/mL) or poly(I:C) (100 ng/mL). Protein expression for ACE2, TMPRSS2 and ADAM17 was investigated after 24 h stimulation and normalized by α-tubulin (n=6). One-way ANOVA followed by Dunnett's multiple comparisons test were used for statistical analysis. Statistically significant p-values are shown. NS – Not Significant.

Supplemental Figure S8. Phosphorylation of STAT1 and STAT2 induced by IFNs in microvascular endothelial cells. Microvascular endothelial cells (MEC) were treated with IFNλ3 (100 ng/mL) or IFNα (100 IU/mL). Protein expression was investigated after 24 h stimulation for (A) p-STAT1 (Tyr701) and (B) p-STAT2 (Tyr690). P-STAT1 was normalized by α-tubulin because IFNs changed the expression of total-STAT1. P-STAT2 was normalized

by total-STAT2 (n=7). One-way ANOVA followed by Dunnett's multiple comparisons test were used for statistical analysis. Statistically significant p-values are shown.

Supplemental Figure S9. Phosphorylation of STAT1 and STAT2 induced by IFNs in lymphatic endothelial cells. Lymphatic endothelial cells (LEC) were treated with IFNλ3 (100 ng/mL) or IFNα (100 IU/mL) for 10 and 30 min. Protein expression for (A) p-STAT1 (Tyr701), (B) p-STAT2 (Tyr690), (C) p-ERK1/2 (Thr202/Tyr204) and (D) p-P38 (Thr180/182) was investigated by western blotting and normalized by total proteins (n=7). One-way ANOVA followed by Dunnett's multiple comparisons test were used for statistical analysis. Statistically significant p-values are shown. NS – Not Significant.

Supplemental Figure S10. IFNLR1 silencing RNA in microvascular endothelial cells. Microvascular endothelial cells (MEC) were treated with 20 nmol/L of IFNLR1 (IL28RA) siRNA or control siRNA (Scramble – Scr). Expression of IFNLR1 (IFNλ3 receptor) was assessed by flow cytometry after 48 h. Statistically significant p-values are shown. NS – Not Significant.

Supplemental Figure S11. Effects of Spike protein S1 of the SARS-CoV-2 (SP1) on ISGs expression in STAT1 and INFLR1 deficient cells. (A) A595 cells WT and STAT1-KO and (B) A595 cells WT and IFNLR1-KO were stimulated with spike 1 protein (S1) (1 µg/mL). Gene expression for ISG15, IFIT1, and MX1 was investigated after 5 h stimulation and normalized by GAPDH (n=7-9). Group analyses were determined by One-way ANOVA followed by Dunnett's multiple comparisons test. Statistically significant p-values are shown. NS – Not Significant.

Supplemental Figure S12. Production of sVCAM-1, sICAM-1, ET-1 and Angpt2 by microvascular endothelial cells. Microvascular endothelial cells (MEC) were treated with IFNλ3 (100 ng/mL) or IFNα (100 IU/mL) for 24 h. (A) Soluble VCAM (sVCAM), (B) soluble ICAM (sICAM), (C) Endothelin-1 (ET-1) and (D) angiopoietin 2 (Angpt2) were determined in the cell supernatant using multiplex assay (n=7). One-way ANOVA followed by Dunnett's multiple comparisons test were used for statistical analysis. Statistically significant p-values are shown. NS – Not Significant.

Supplemental Figure S13. MCP-1 and IL-6 induced by IFNs in endothelial cells. Primary cultures of human (A) lymphatic endothelial cells (LEC), (B) aortic EC (AEC) and (C) pulmonary EC (PEC) were treated with IFNλ3 (100 ng/mL), IFNα (100 IU/mL) or poli(I:C) (100 ng/mL) for 24 h. MCP-1 and IL-6 were investigated in the cell supernatants by ELISA (n=7). One-way ANOVA followed by Dunnett's multiple comparisons test were used for statistical analysis. Statistically significant p-values are shown. NS – Not Significant.

Supplemental Figure S14. Vascular function in vessels from WT normotensive and LinA3 hypertensive mice. Small mesenteric arteries (the vessels important in peripheral resistance and hypertension) were isolated from wild type (WT) (circles, n=8) and hypertensive LinA3 (squares, n=6) mice and investigated for (A) acetylcholine (Ach)-relaxation, (B) U46619-contraction and (C) sodium nitroprusside (SNP). Vessels from (D-G) WT and (H-K) LinA3 mice were mounted on wire myographs and treated with IFNλ3 (100 ng/mL, blue), IFNα (100 IU/mL, red) for 1 h or SP1 (1μg/mL, green) for 2 h, followed by concentration-response curves to (main figure 7) acetylcholine (Ach)-relaxation, (D, F, H, J) U46619-contraction and (E, G, I, K) sodium nitroprusside (SNP). U46619 tension curves are expressed in mN. Ach and SNP curves are expressed in % of relaxation compared to pre-constriction induced by U44619. Vascular function, maximal response (Emax) values from experimental data were fitted to a four-parameter logistic function against the null hypothesis and two-way ANOVA followed by Bonferroni's post-test for multiple comparisons. Statistically significant p-values are shown. NS – Not Significant.

Supplemental Figure S15. Vascular function in vessels from ISG15+/+ and ISG15-/- mice. Small mesenteric vessels were isolated from ISG+/+ (circles, n=8) and ISG-/- (squares, n=6) mice and investigated for (A) acetylcholine (Ach)-relaxation and (B) U46619-contraction. Vessels from (C, E) ISG+/+ and (D, F) ISG15-/- mice were mounted on wire myographs and treated with IFNλ3 (100 ng/mL, blue), IFNα (100 IU/mL, red) for 1 h or SP1 (1μg/mL, green) for 2 h, followed by concentration-response curves to (C, D) U46619-contraction and (E, F) acetylcholine (Ach)-relaxation. U46619 tension curves are expressed in mN. Ach curves are expressed in % of relaxation compared to pre-constriction induced by U44619. Vascular function, maximal response (Emax) values from experimental data were fitted to a four-parameter logistic function against the null hypothesis and two-way ANOVA

followed by Bonferroni's post-test for multiple comparisons. Statistically significant p-values are shown. NS – Not Significant.

Supplemental Figure S1

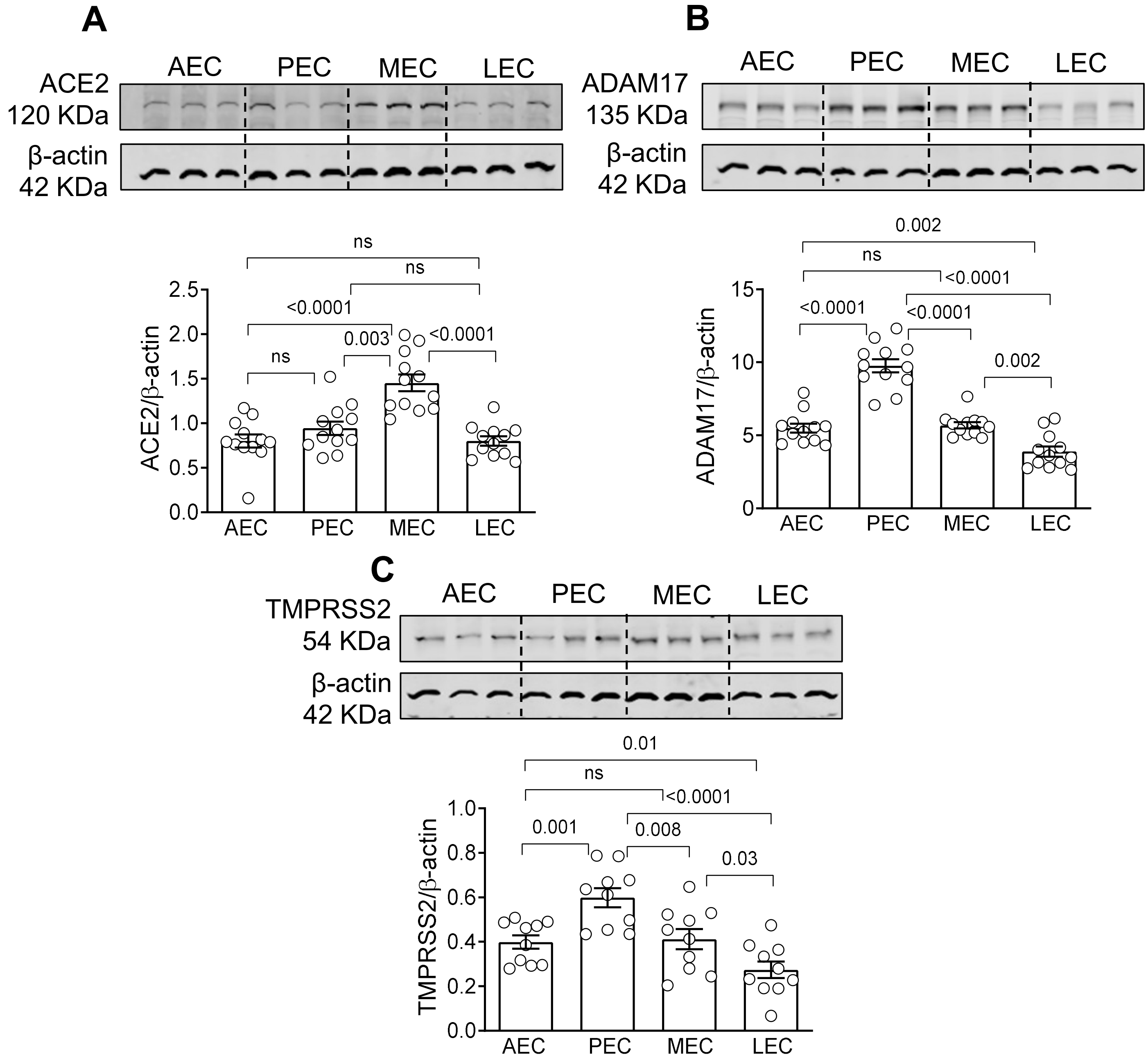

Supplemental Figure S2

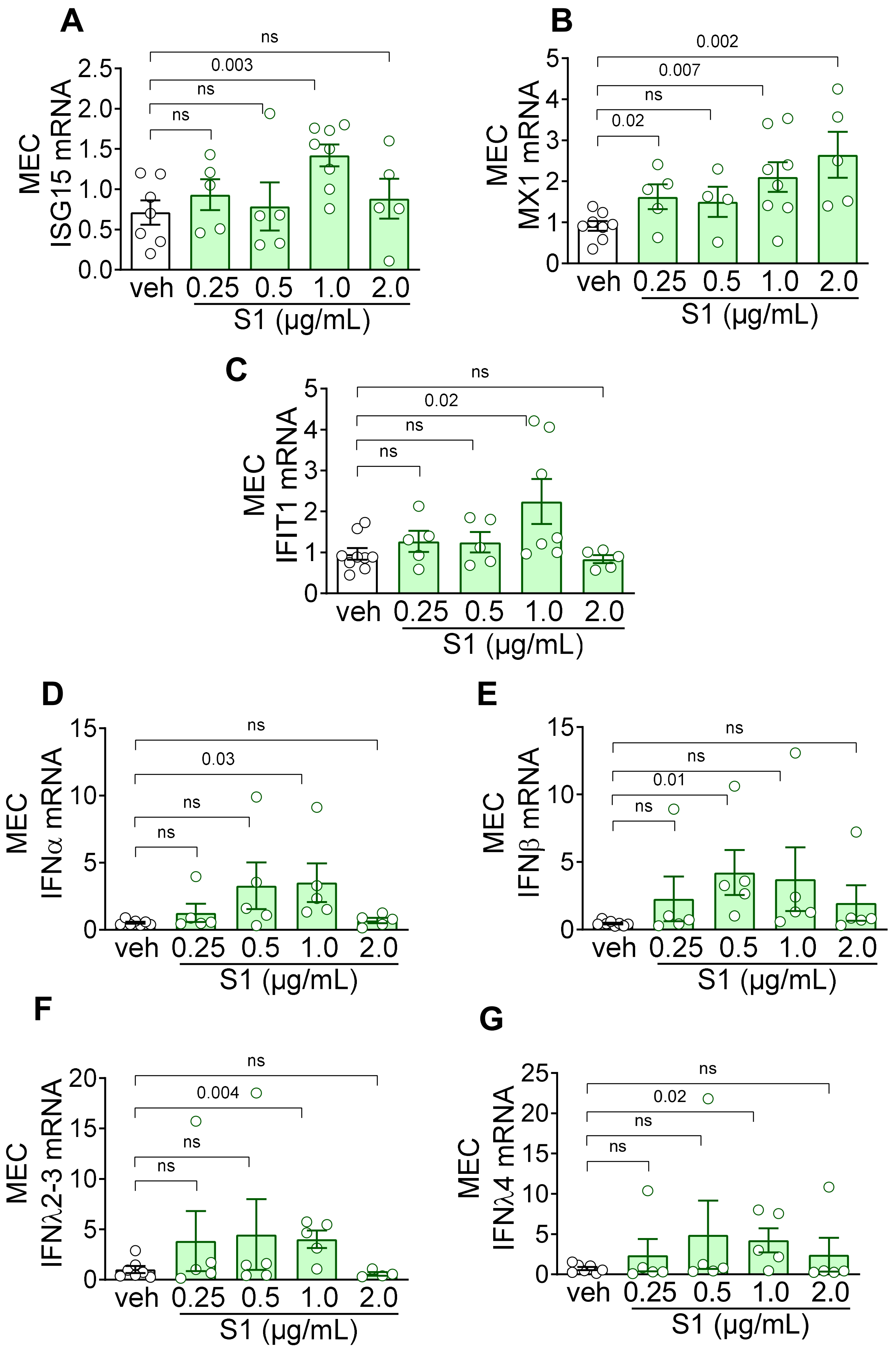

Supplemental Figure S3

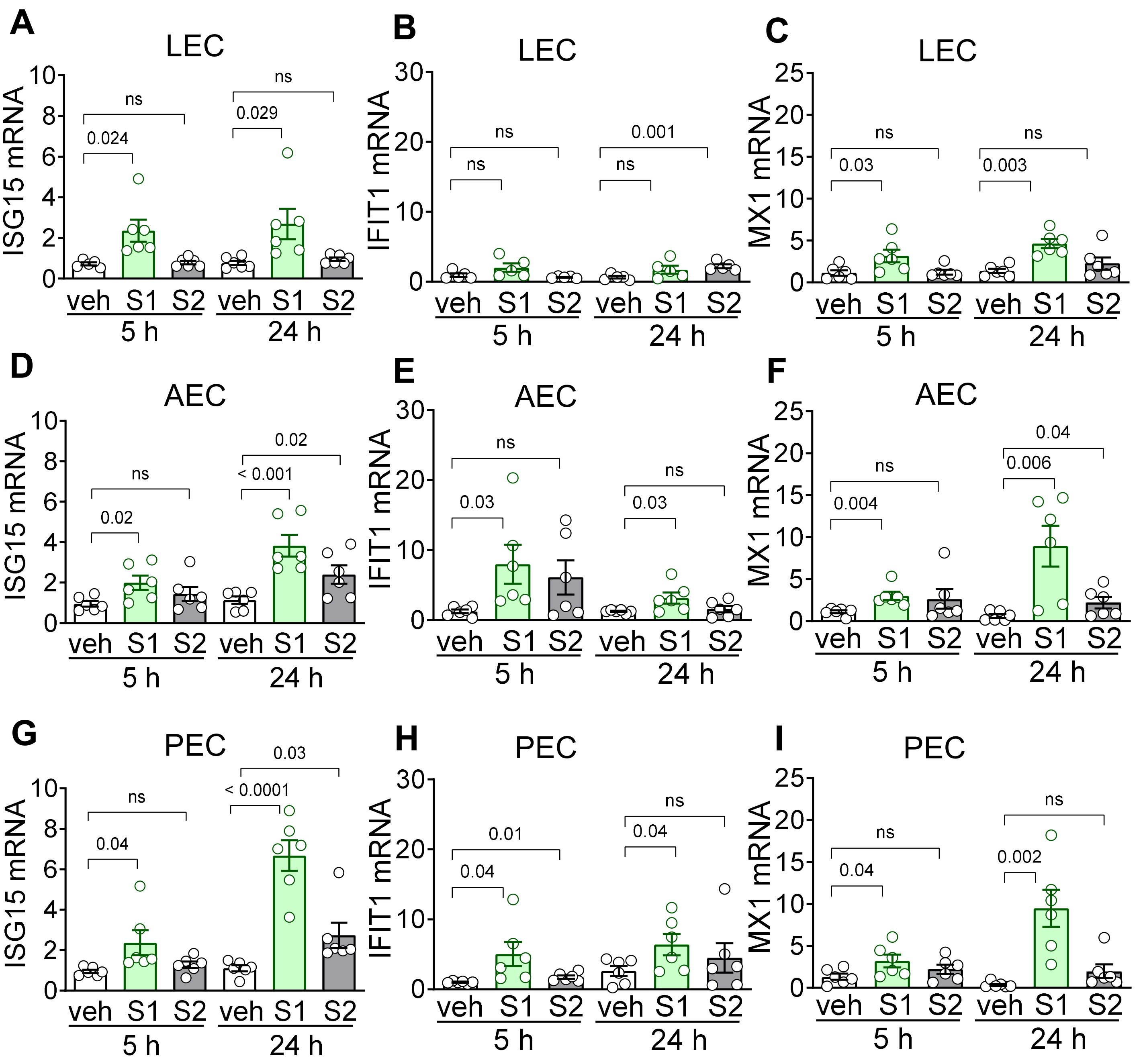

Supplemental Figure S4

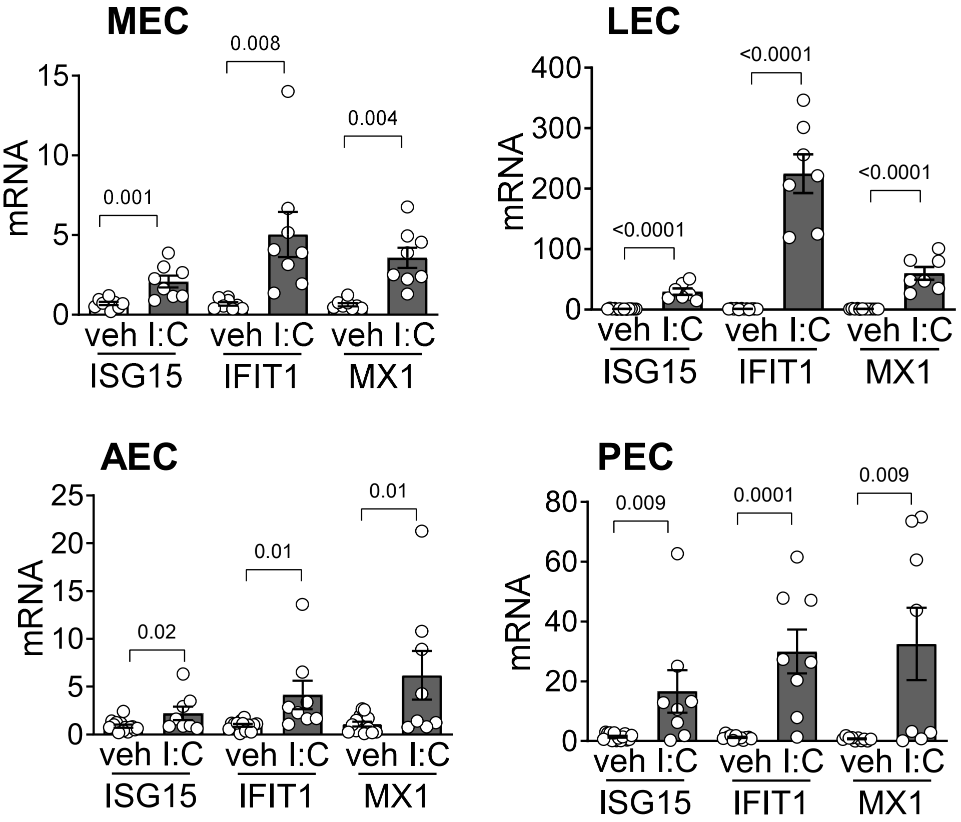

Supplemental Figure S5

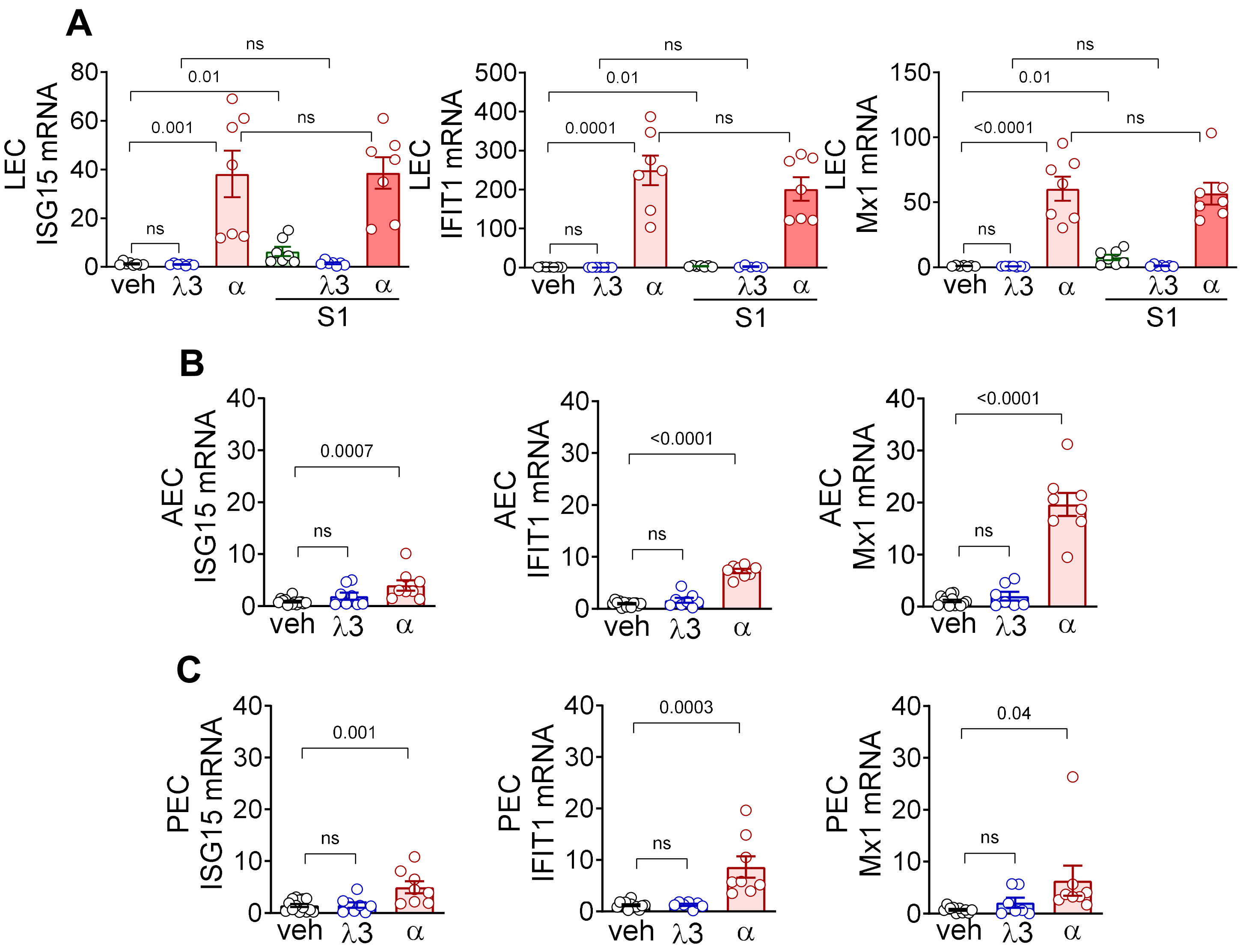

Supplemental Figure S6

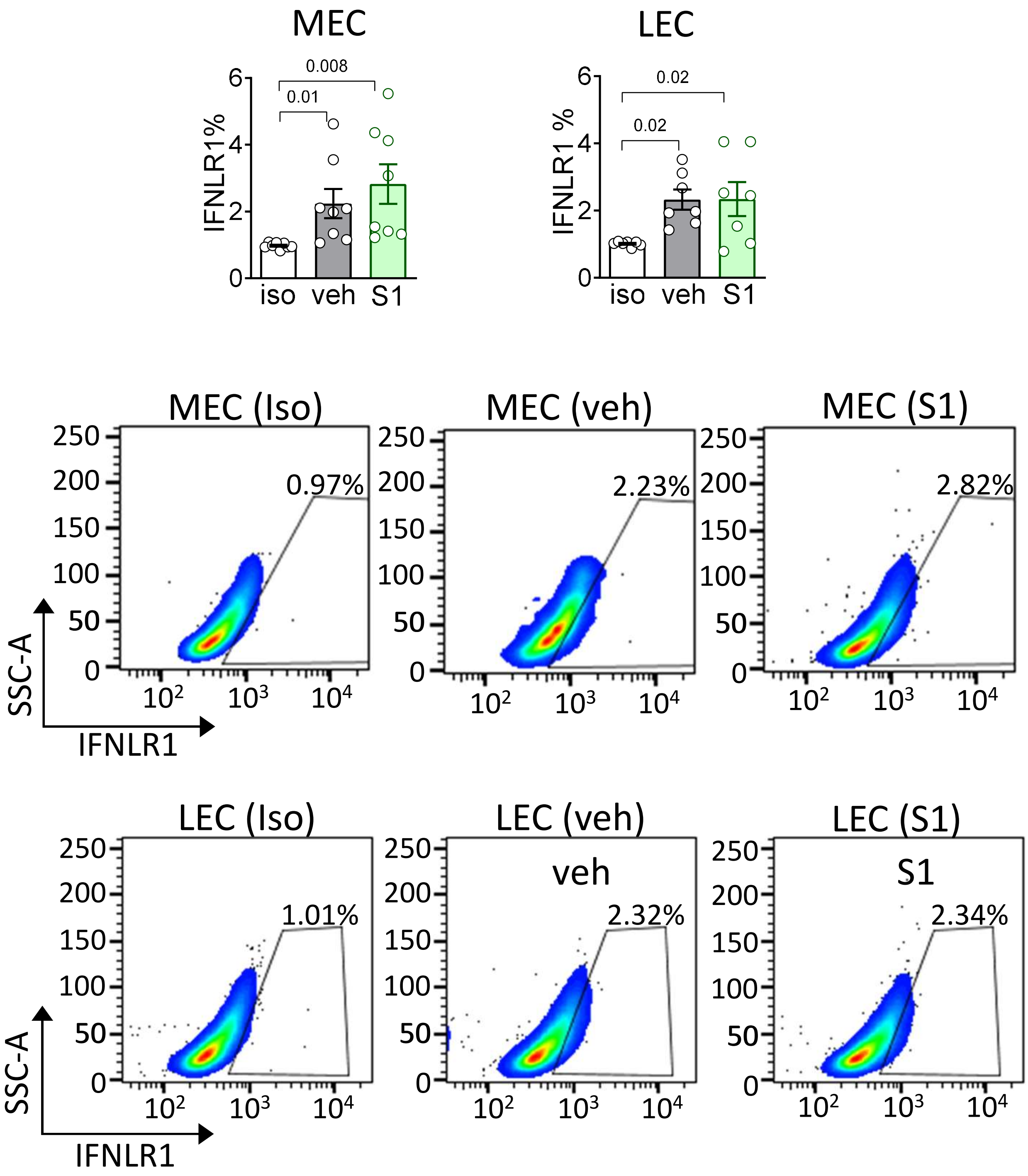

Supplemental Figure S7

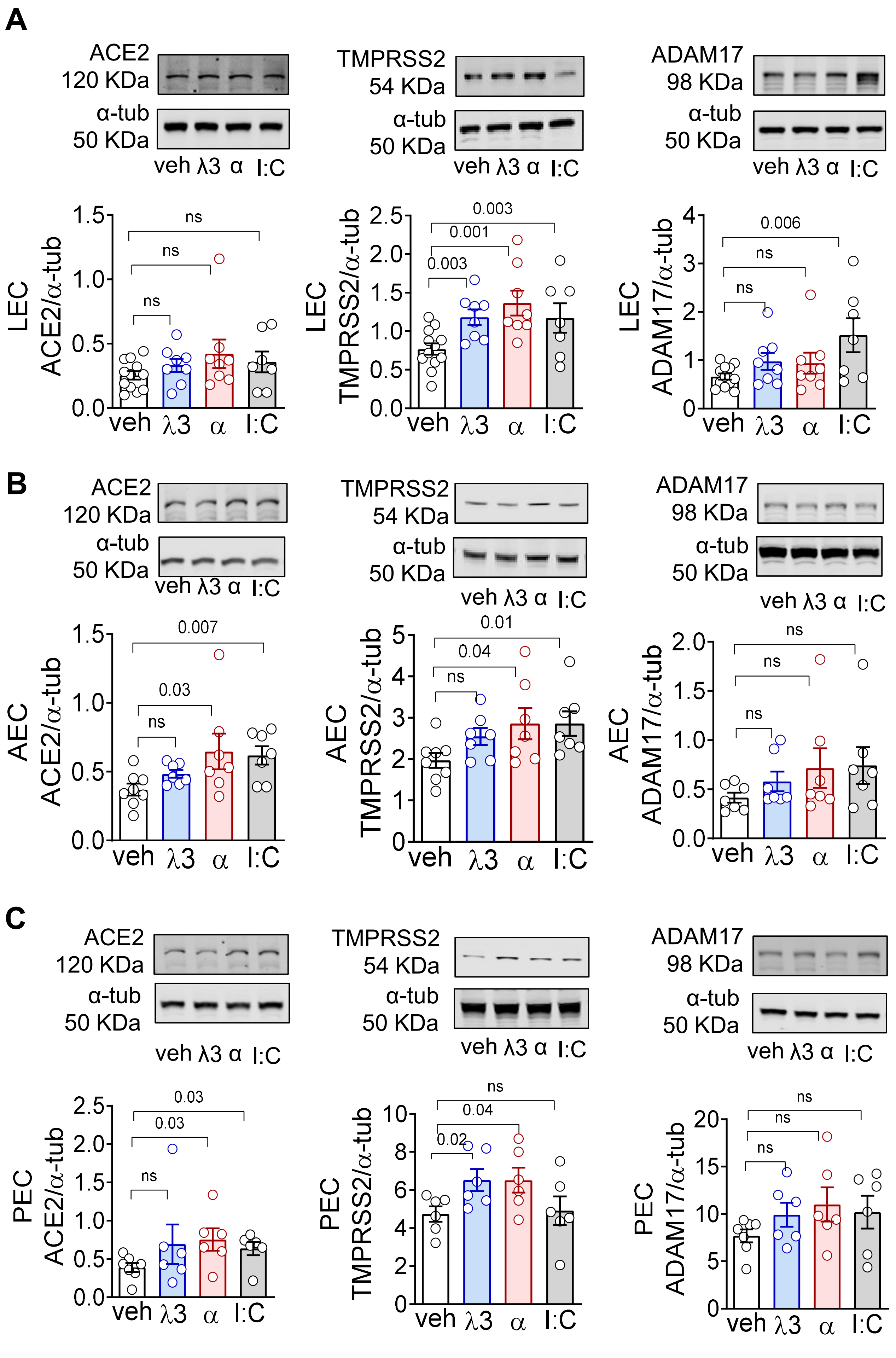

Supplemental Figure S8

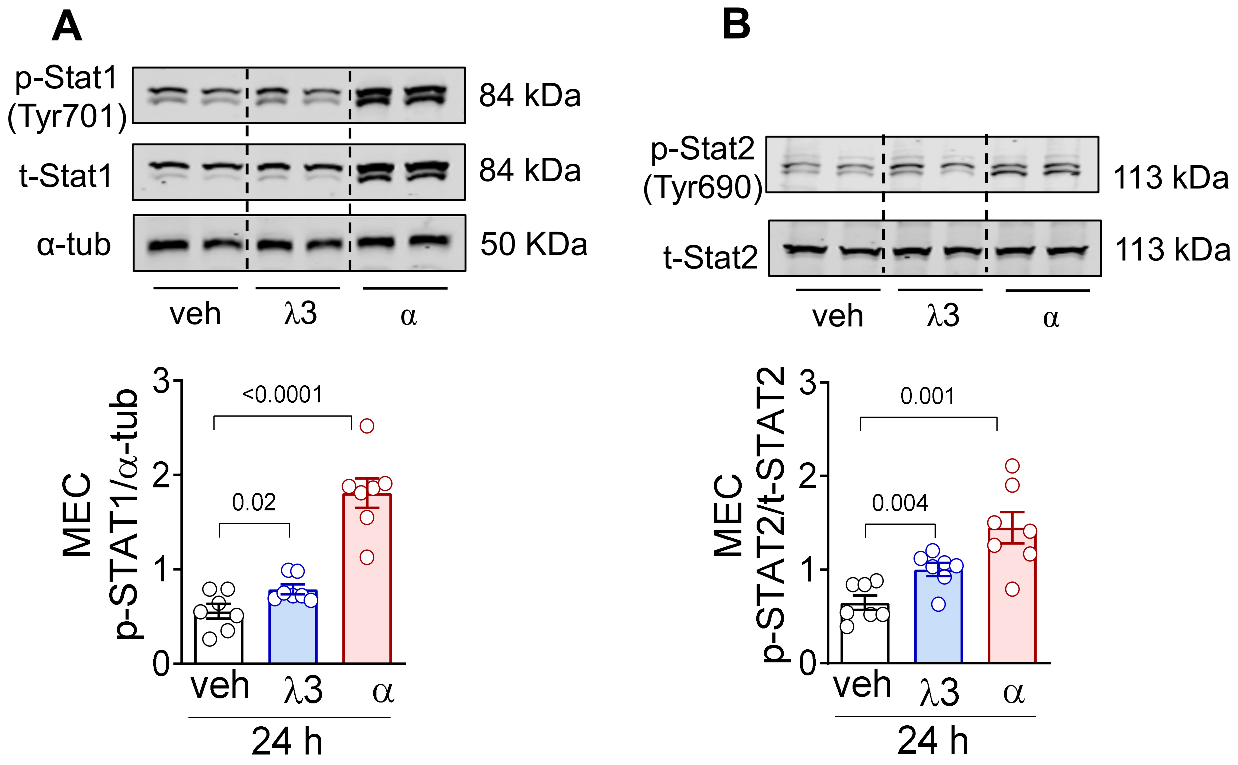

Supplemental Figure S9

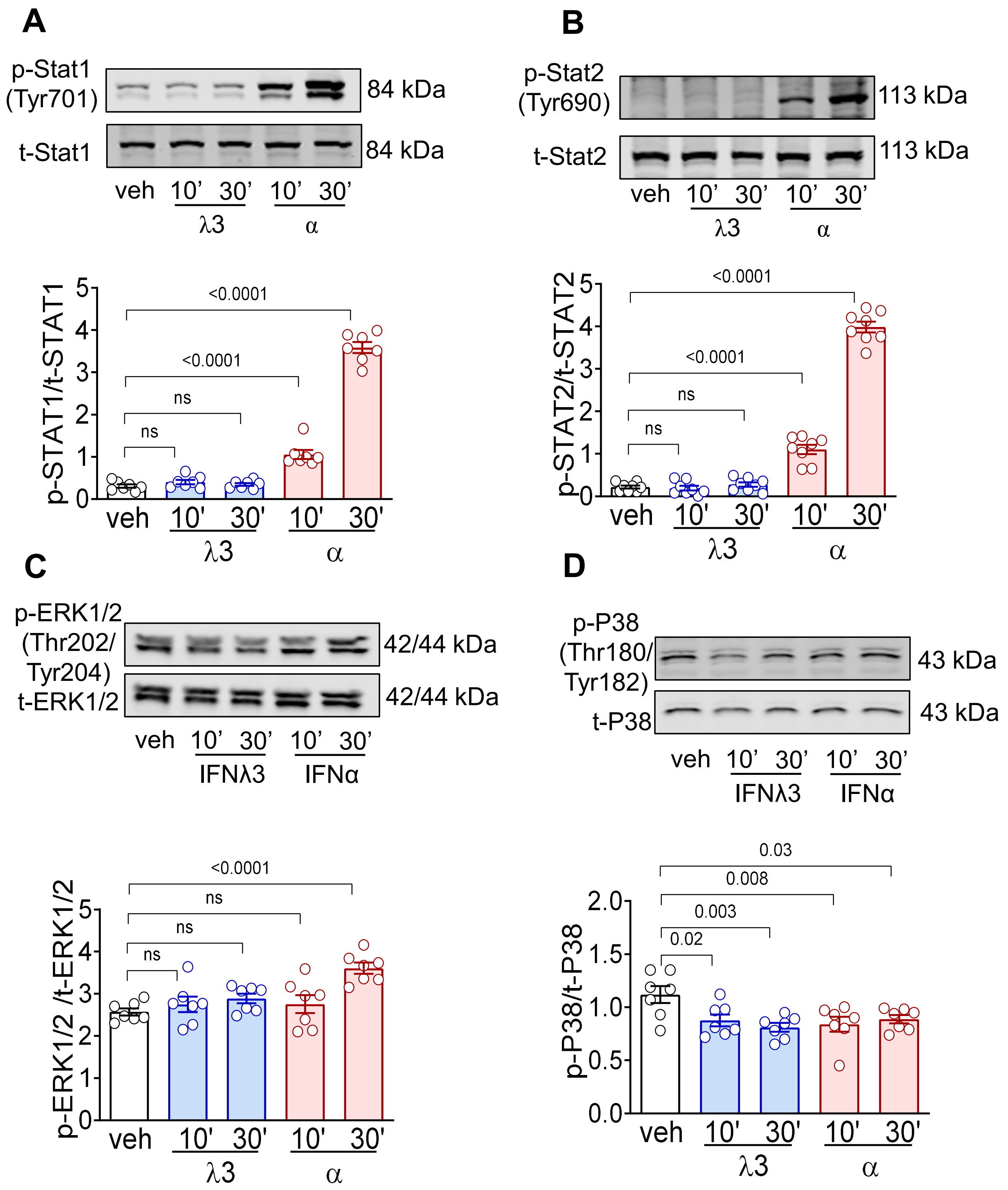

Supplemental Figure S10

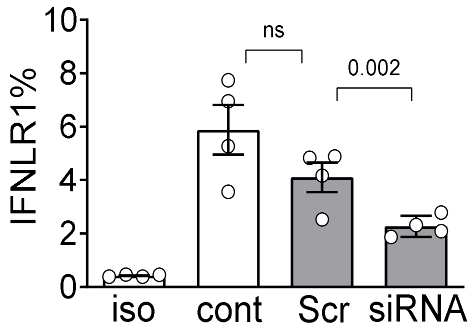

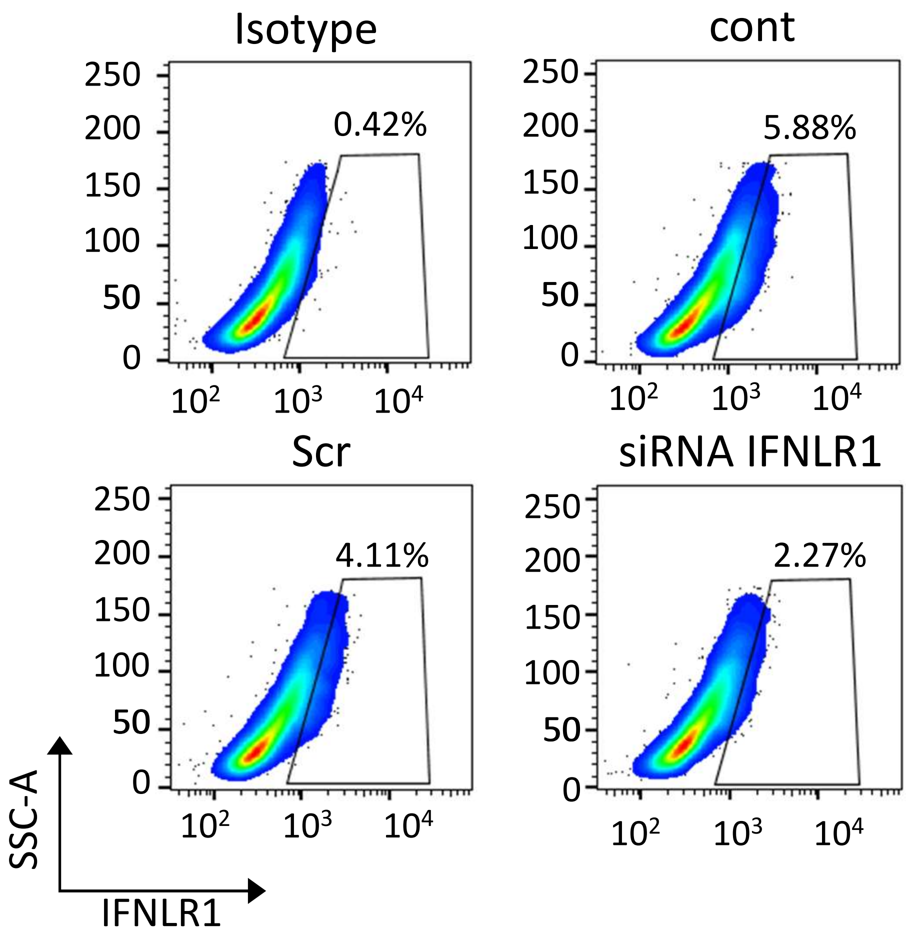

Supplemental Figure S11

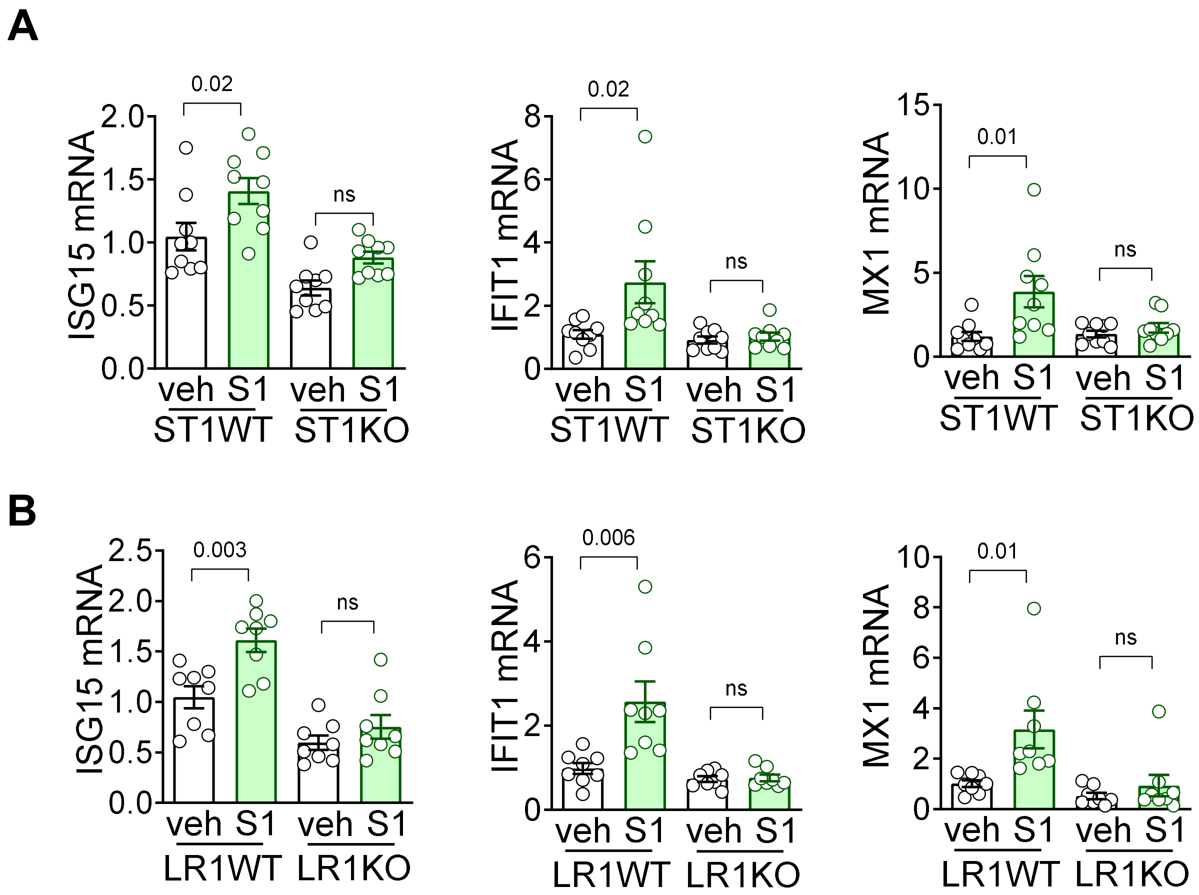

Supplemental Figure S12

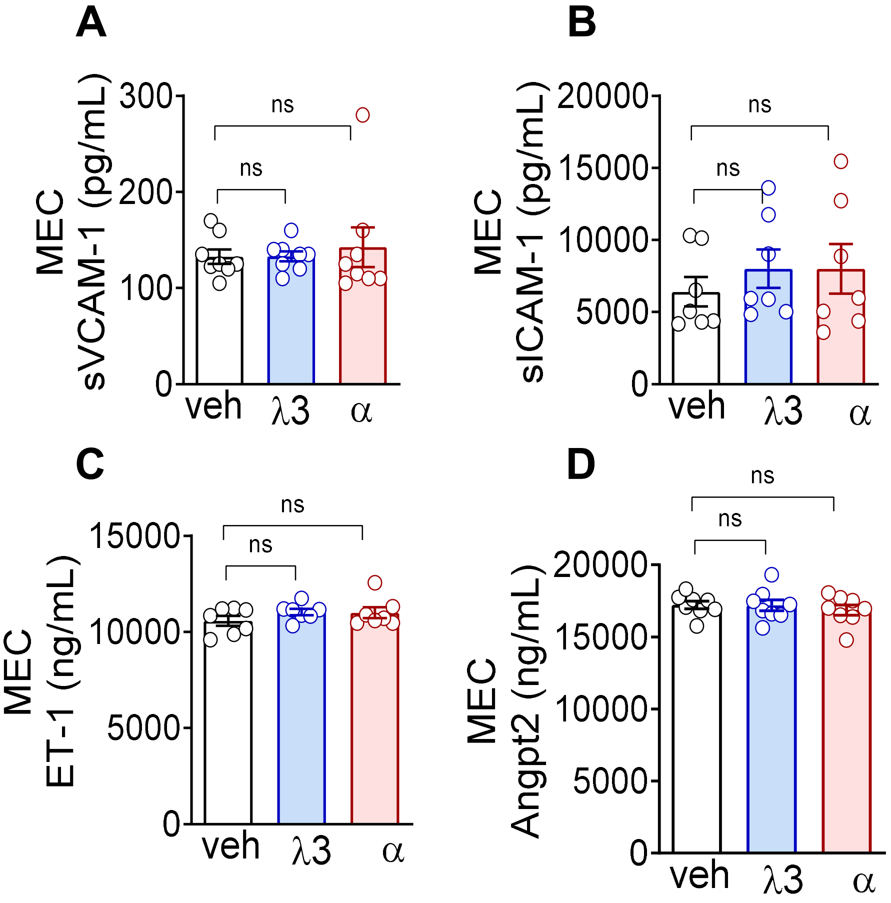

Supplemental Figure S13

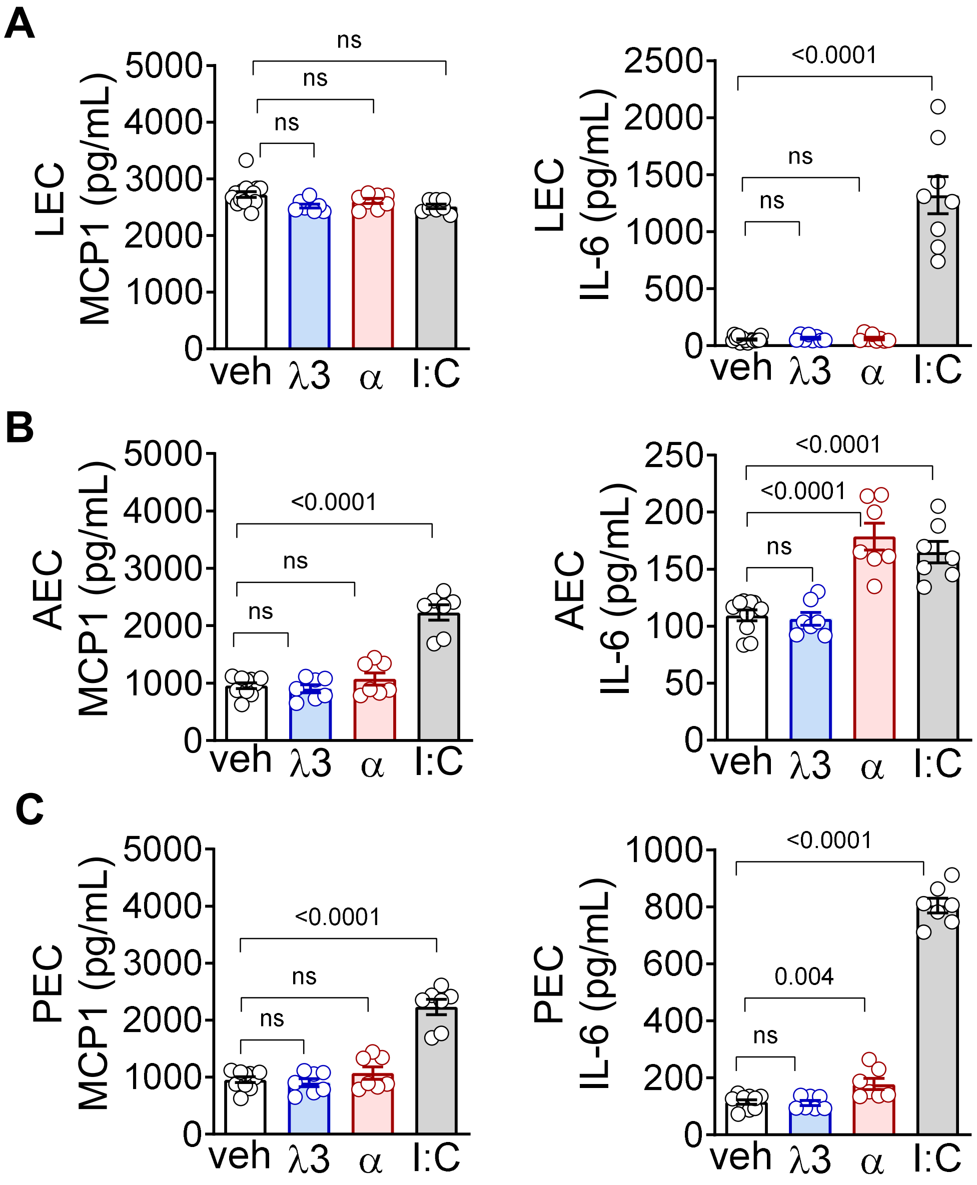

Supplemental Figure S14

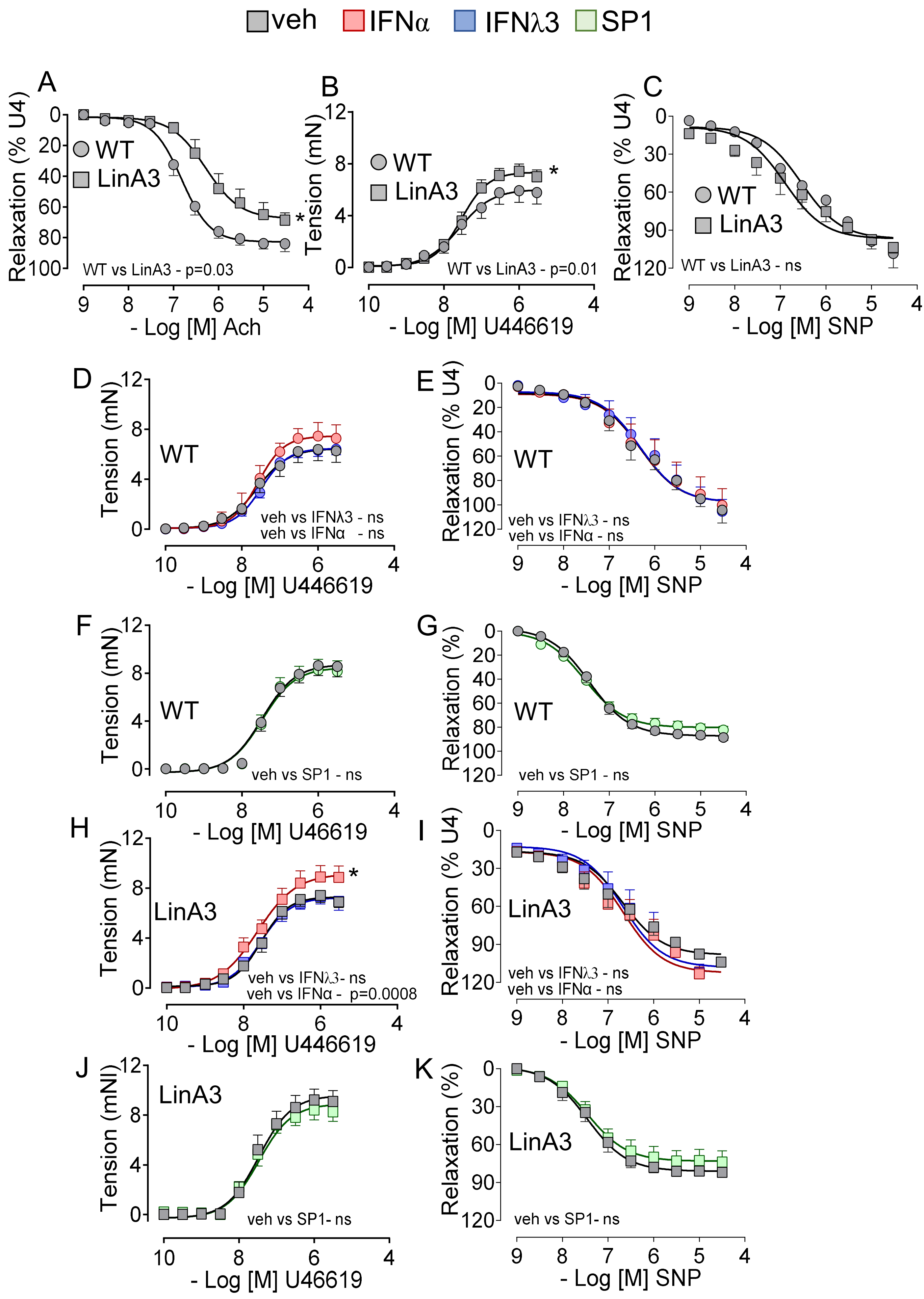

Supplemental Figure S15

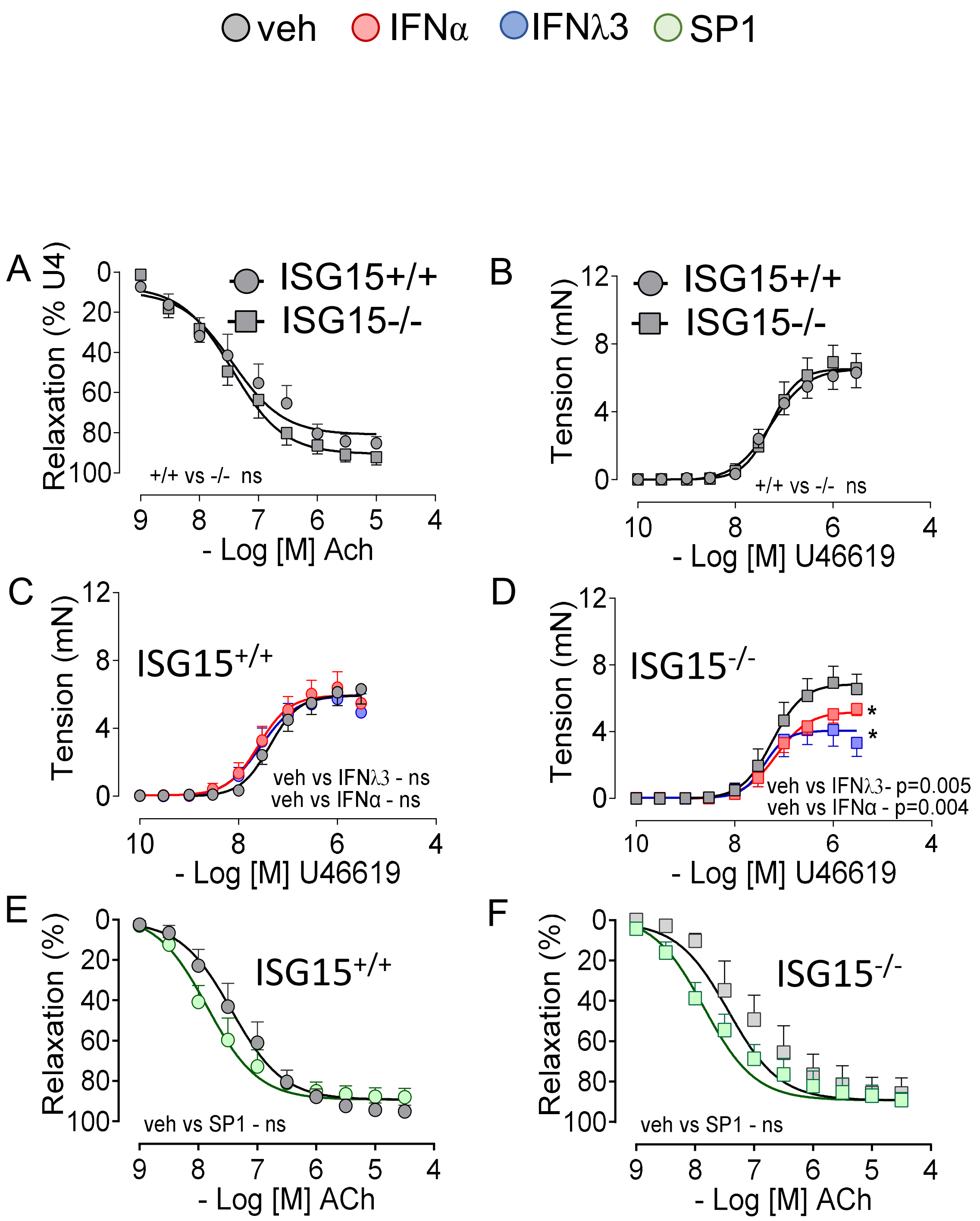

Full gels of the main figures

Red arrow indicates protein of interest

Figure 2D - PCNA

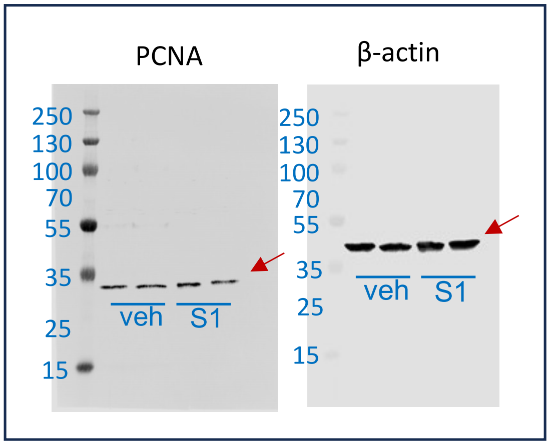

Figure 3

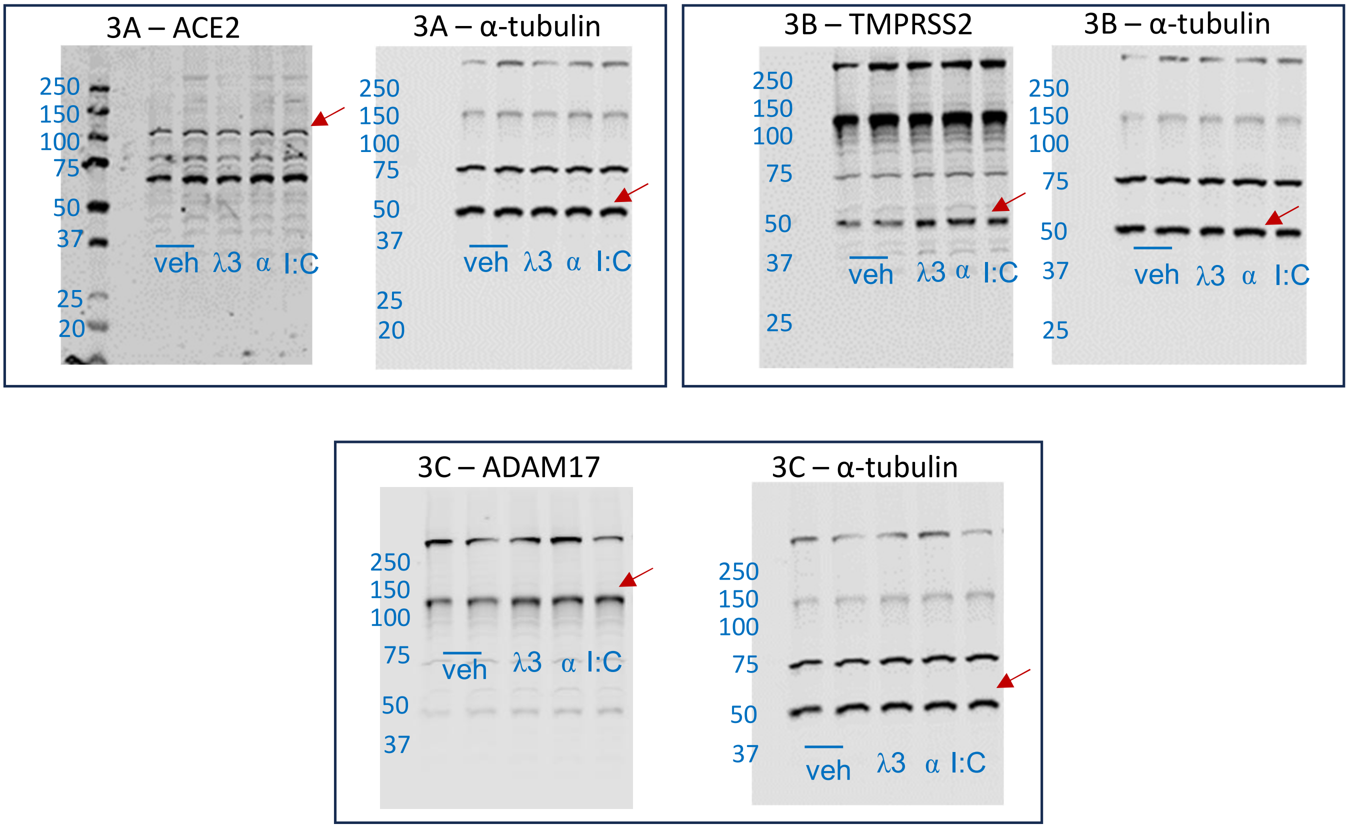

Red arrow indicates protein of interest

Figure 4A

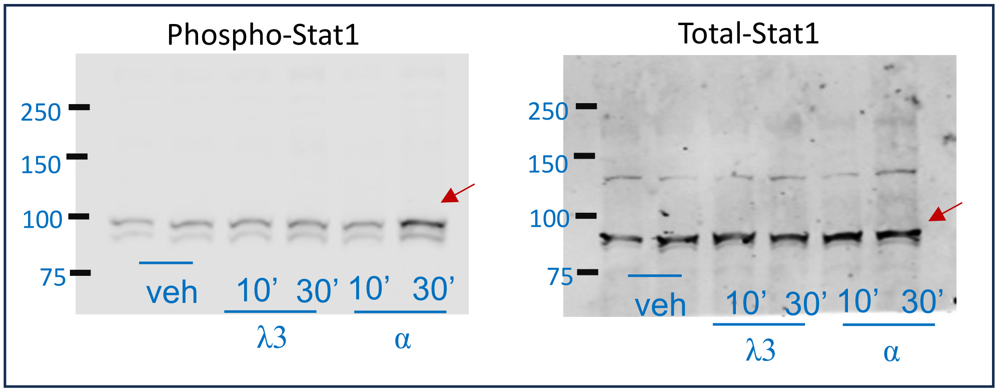

Figure 4B

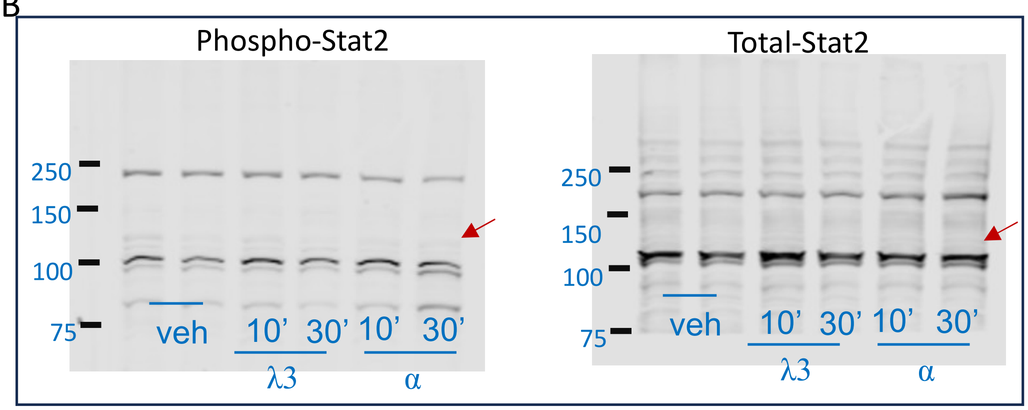

Figure 4C

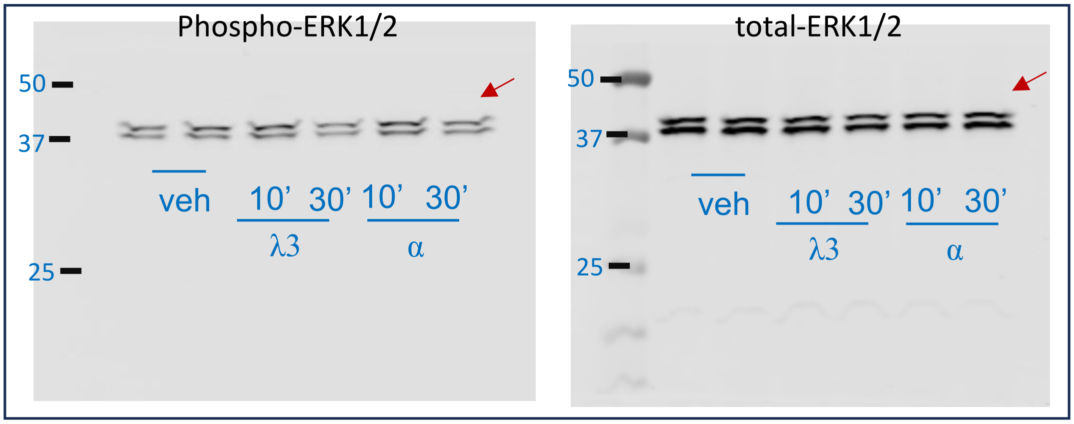

Figure 4D

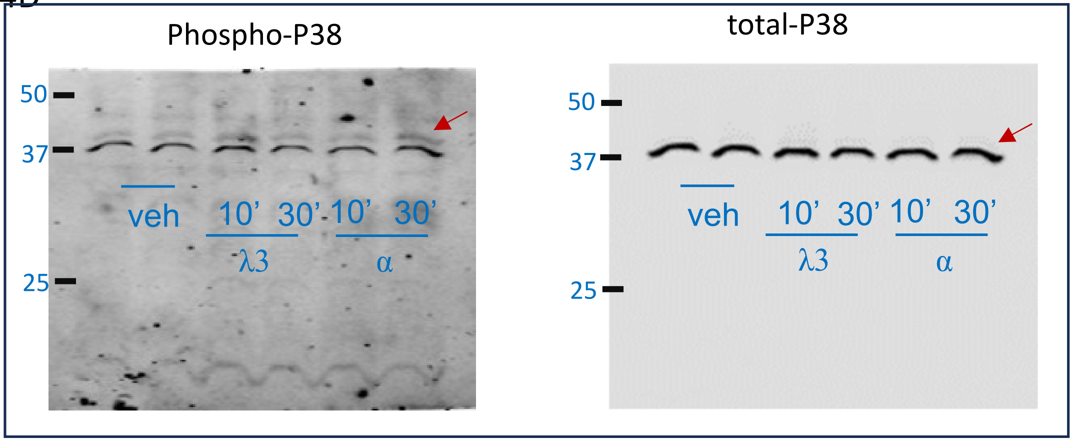

Red arrow indicates protein of interest

Figure 6B

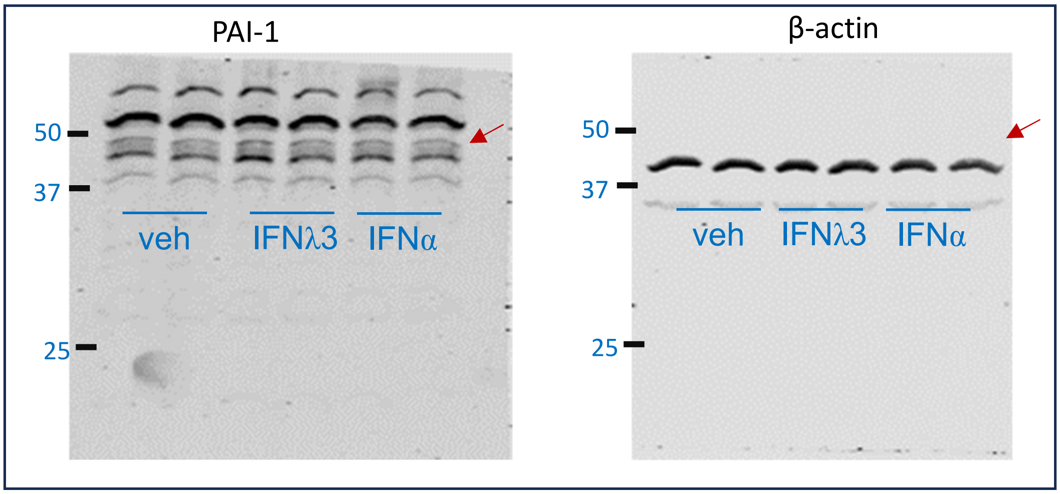

Figure 6D

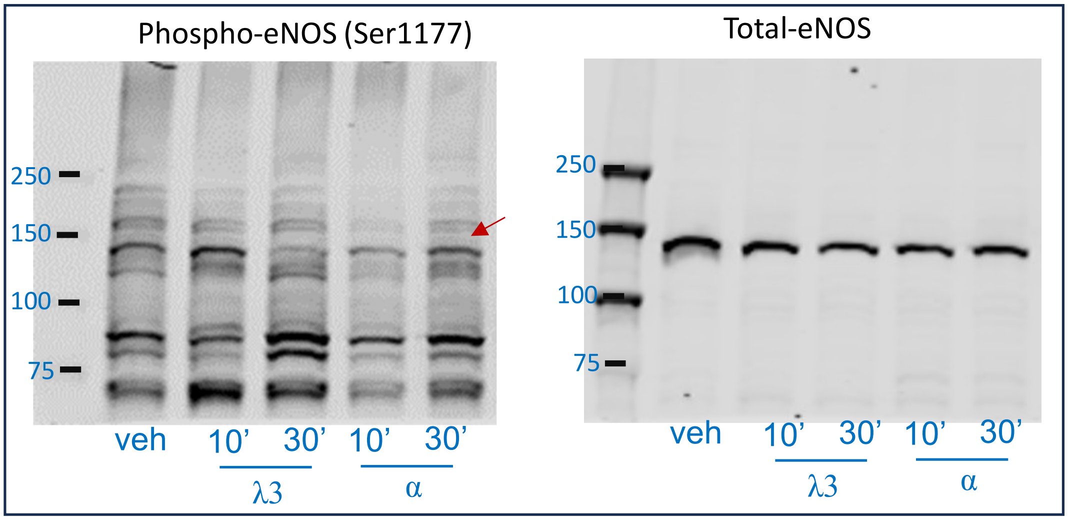

Figure 6E

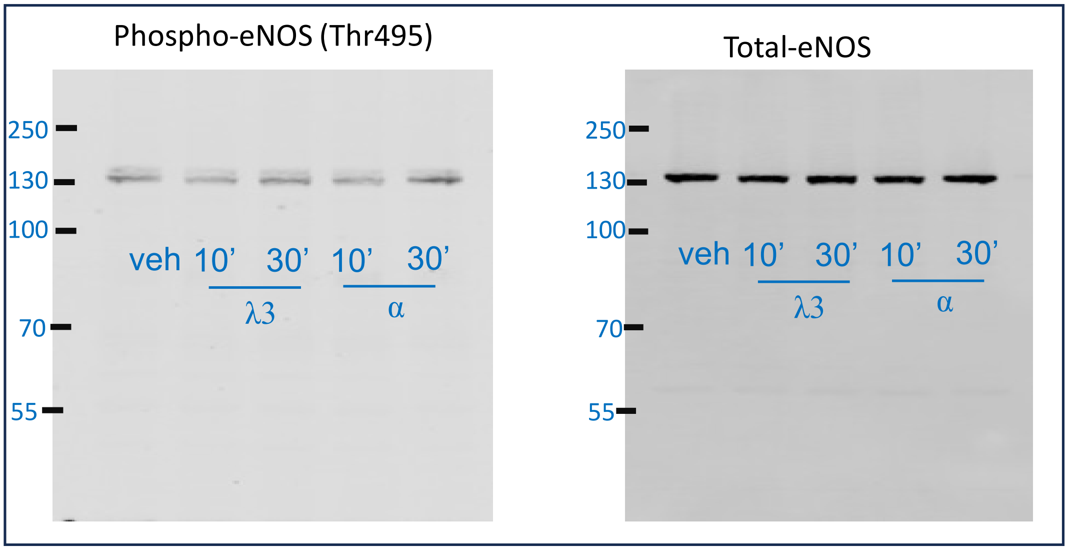

Full gels - Supplemental Figure S1

Red arrow indicates protein of interest

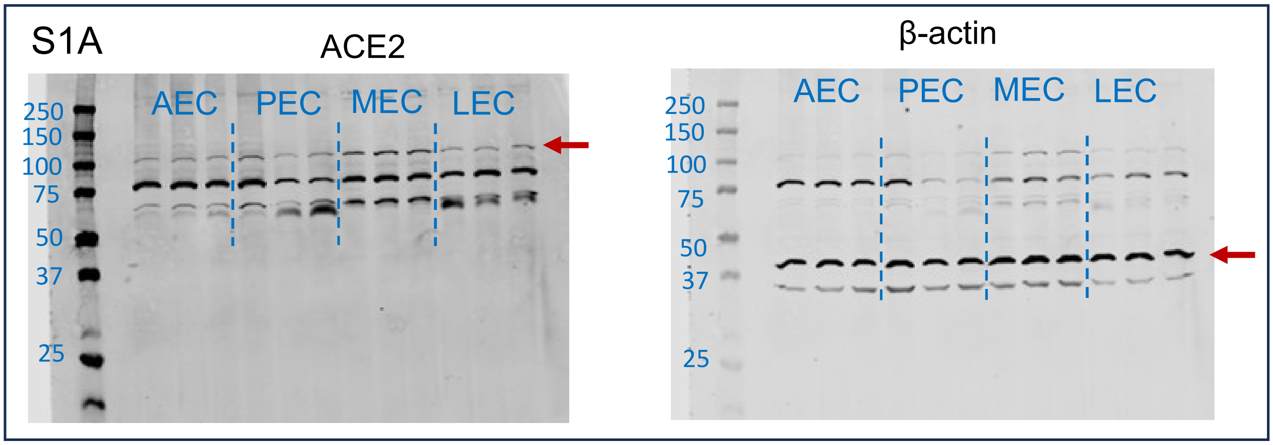

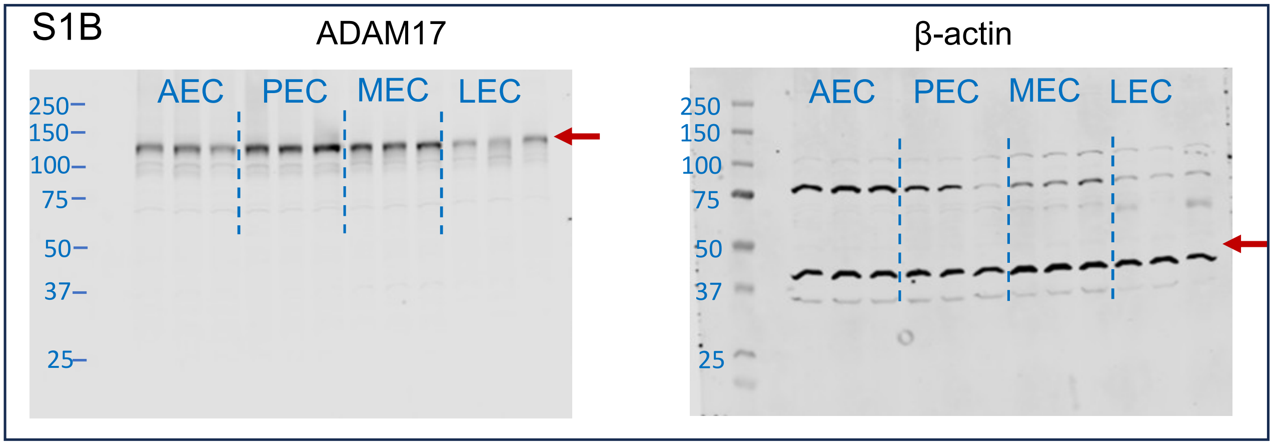

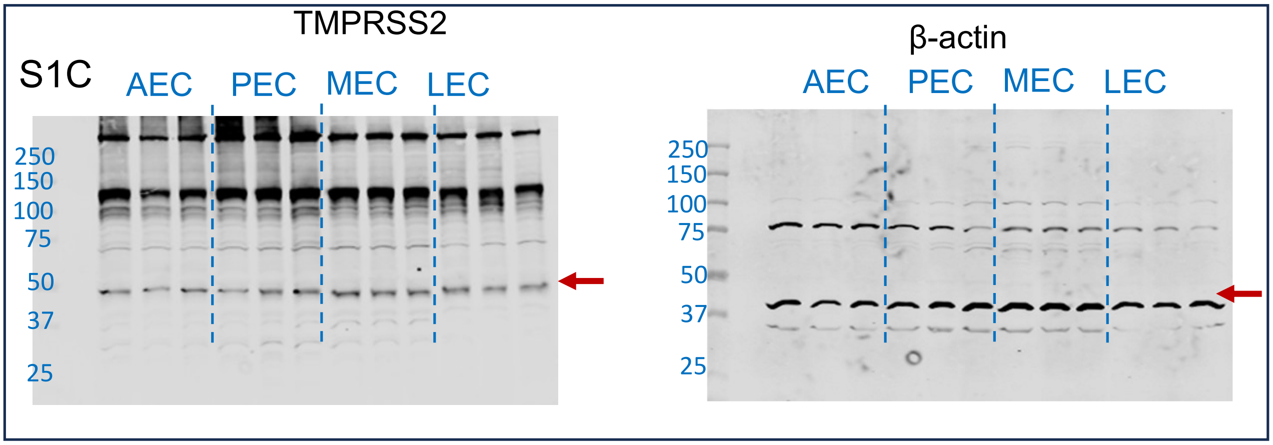

Full gels - Supplemental Figure S7

Red arrow indicates protein of interest

S7A

LEC

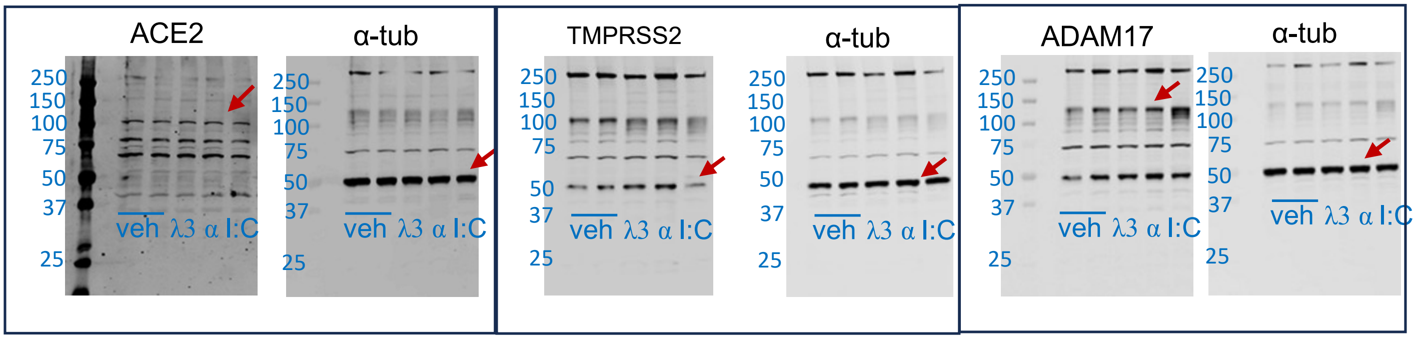

S7B

AEC

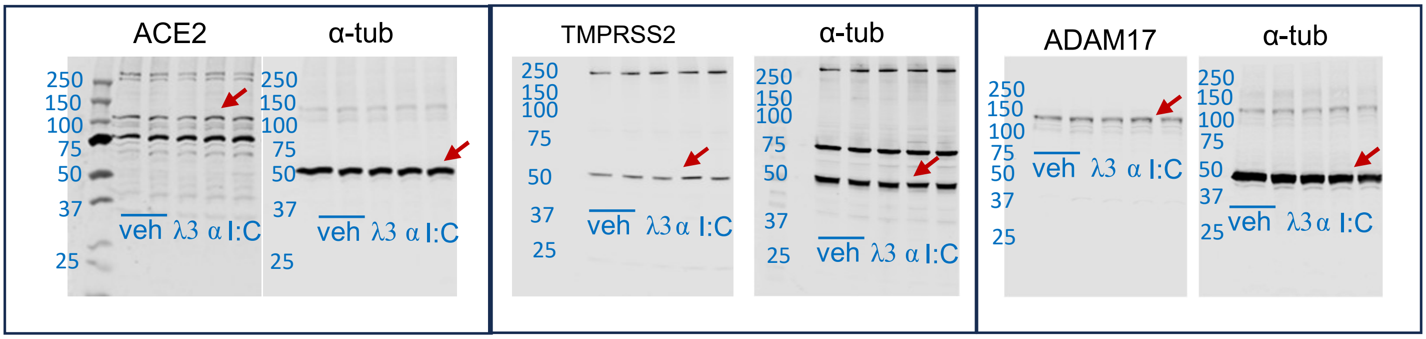

S7C

PEC

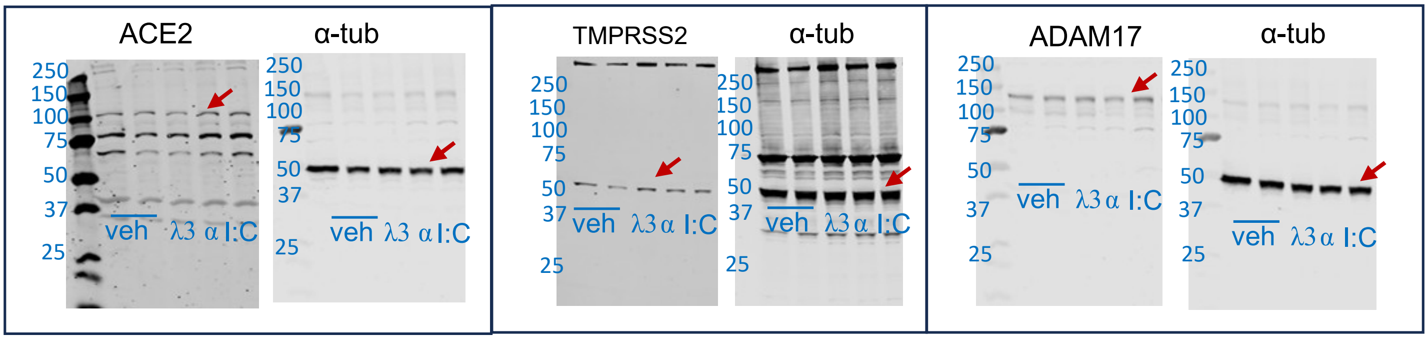

Supplemental Figure S8

Red arrow indicates protein of interest

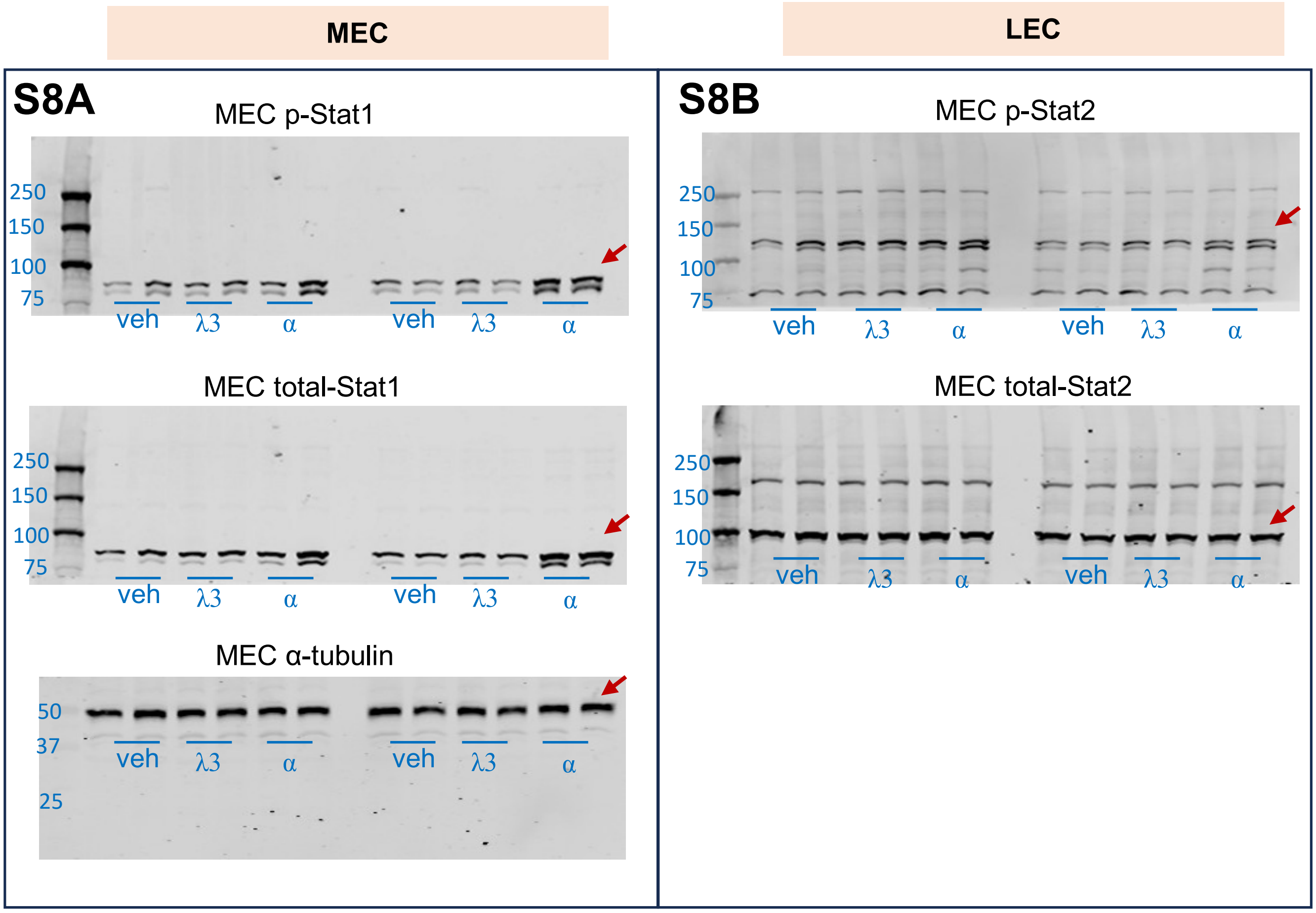

Supplemental Figure S9

Red arrow indicates protein of interest

LEC

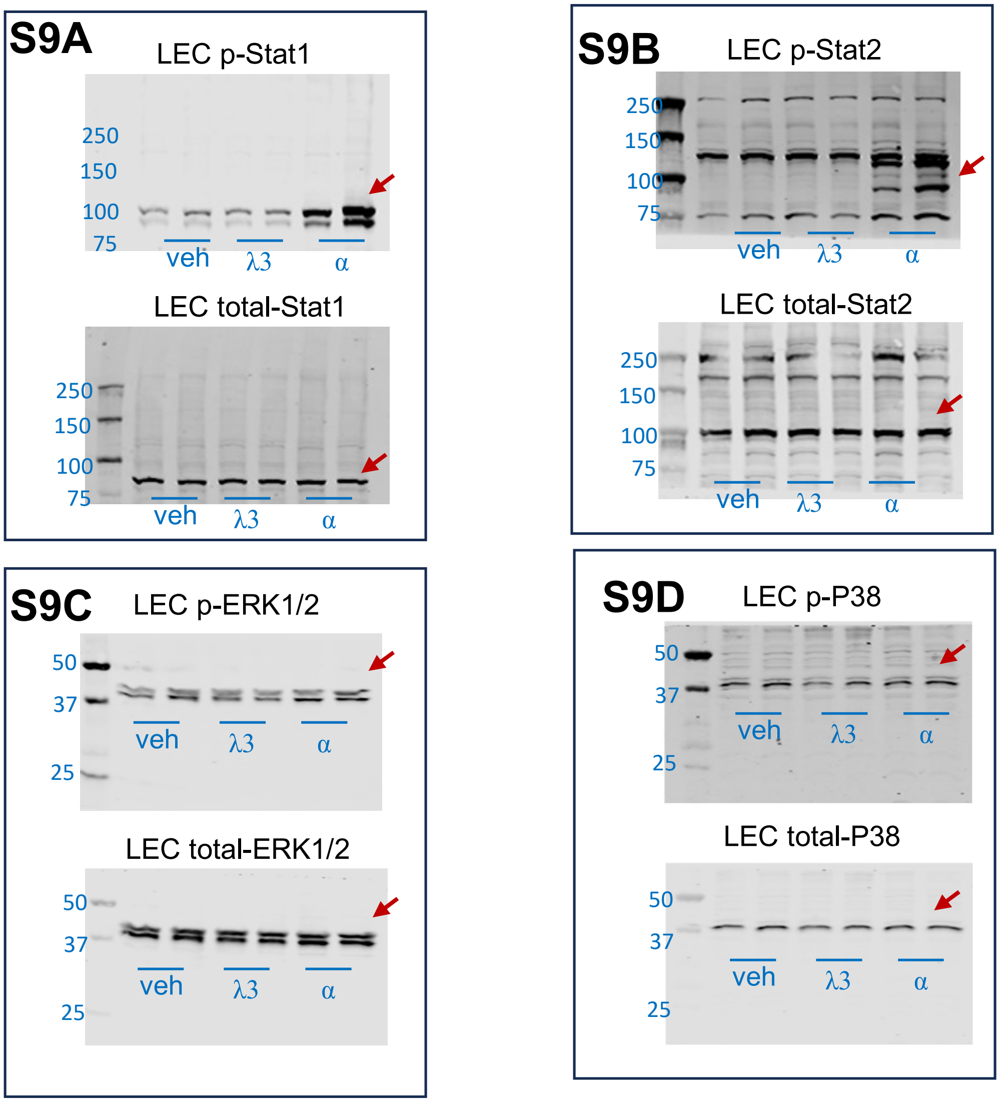

Supplement: cvag111_Supplementary_Data [file cvag111_supplementary_data.pdf]
